# Evaluation of morphological traits, biochemical parameters and seeding availability pattern among *Citrus limon* ‘Assam lemon’ accessions across Assam

**DOI:** 10.1038/s41598-024-54392-3

**Published:** 2024-02-16

**Authors:** Suraiya Akhtar, Raja Ahmed, Khaleda Begum, Ankur Das, Sarat Saikia, Rafiul Amin Laskar, Sofia Banu

**Affiliations:** 1 Department of Bioengineering and Technology, Gauhati University, Guwahati, Assam 781014 India; 2 Horticulture Research Station, Assam Agricultural University, Kahikuchi, Guwahati, Assam 781017 India; 3 Department of Botany, Pandit Deendayal Upadhyaya Adarsha Mahavidyalaya (PDUAM), Eraligool, Karimganj, Assam 788723 India

**Keywords:** *Citrus limon*, Assam lemon, Biochemical variation, Morphological variation, Seeding pattern, Soil analysis, Evolution, Plant sciences

## Abstract

The Assam lemon is a highly valued *Citrus* cultivar known for its unique aroma, flavor, and appearance. This study aimed to investigate the morphological, seeding pattern and biochemical variations within 132 populations of Assam lemon from across 22 districts of Assam along with the control samples, with the objective to offer comprehensive understanding that could facilitate the improvement of breeding programs and further improvement of this important cultivar. Clustering based on UPGMA algorithm for morphological and seeding pattern data were analysed at population level, revealed two major clusters, where all the populations of Upper Assam districts were in the same cluster with the original stock (control population). The populations from Tinsukia and Dhemaji districts displayed more close similarities with the control population in comparison to populations of Upper Assam districts. Another interesting observation was regarding flowering patterns, while populations from Upper Assam districts excluding Golaghat district displayed both bisexual and unisexual flowers with less concentration of unisexual flowers, other remaining districts had bisexual and unisexual flowers of almost equal concentration. Unisexual flowers contained only the male reproductive organs with 40 anthers, while bisexual flowers had 36 anthers. Seeding patterns were examined across the districts, and it was found that populations from Tinsukia, Dhemaji, Lakhimpur, Dibrugarh, Jorhat, and the control population exhibited seedless characteristic while populations from other selected districts displayed a combination of seedless and seeded traits. Interestingly, Golaghat district appears as the linking district and showed availability of both seeded and seedless Assam lemon fruit, connecting the regions of Barak valley, Central, Lower, North and Upper Assam. Biochemical analysis showed significant variations across districts, however, the populations from Dhemaji, Tinsukia, Lakhimpur, Dibrugarh, and Jorhat districts displayed similarity with the control population. The study also investigated variability in soil nutrient content revealing substantial variation among the populations studied. This comprehensive investigation provides valuable insights into the morphological, seeding pattern, and biochemical diversity within the Assam lemon cultivar. These findings can be instrumental in breeding programs to enhance the cultivar, particularly in producing high-quality seedless fruits to meet consumer demands.

## Introduction

*Citrus* fruits are globally renowned for their economic importance, nutritional value, and cultural significance^1^. In India, Assam stands as a prominent region for *Citrus* cultivation, encompassing diverse *Citrus* species with distinct flavours, aromas, and appearance^2^. Among this, Assam lemon, an important cultivar of *C. limon* holds a special place as a widely grown lemon cultivar in Assam^3^. Known for its distinct aroma, and flavour, Assam lemon has gained popularity in both domestic and international markets^4^. Further, due to its nutritional value, it is used as source for medicinal and cosmetic products^5^. The morphology of Assam lemon exhibits a range of traits, including fruit size, fruit shape, fruit colour, peel texture, seed numbers etc.^6^. These morphological variations may be influenced by factors such as soil type, rainfall, and agricultural practices followed by farmers in different districts^7^. The understanding of morphological variation within Assam lemon cultivar across districts of Assam is expected to assist in breeding programs for further improvement of Assam lemon^8^.

Assam lemon forayed into the cultivation and market after being identified at Government Citrus Fruit Research Station, Byrnihat^9^. In the 1940s, S.C. Bhattacharyya and S. Dutta, Horticulture Development Officer and Officer-in-charge respectively, of Government *Citrus* Fruit Research Station (GCFRS), Byrnihat, collected a variety from Hahchora village under the name “China-kaghi”^4^. While experimenting with the seeds, they stumbled upon a seedling which was characteristically different from others and had seedless fruit^9^. In order to preserve this unique quality of non-seediness, it was further propagated by vegetative means and developed as a clonal horticultural cultivar and named as Assam lemon as documented in the monograph—Classification of *Citrus* Fruits of Assam, published by the Indian Council of Agricultural Research in 1956^10^. Assam lemon has acquired a prized status as the second most grown *Citrus* variety of Assam with about more than 15 thousand hectares of cultivated area and an annual production of more than 1.56 lakh metric tons^14^. Assam lemon, a lemon like no other, boast an extraordinary aroma, flavour, and seedlessness that set it apart from its counterparts^9^. Farmers, both commercial and domestic, have embraced the lemon’s unique qualities, cultivating it in homestead gardens as a standalone crop or alongside other *Citrus* varieties^9^. *Citrus* species, including Assam lemon, have a tendency towards natural hybridization, which makes it highly labile to acquire variations in seed formation in different populations over time^11,12^. Moreover, study by Kahn et al. (2004) highlighted that specific *Citrus* varieties can yield seedless fruits due to limited functional pollen and ovules^13^. Conversely, some *Citrus* types may possess ample functional pollens and ovules but are self-incompatible, resulting in seedless fruits when grown alone. However, when grown near cross-pollinating varieties, these self-incompatible types can produce seedy fruits^13^. In our previous work, we studied the distribution of genetic diversity of Assam lemon within locations of Assam through ISSR marker analysis and stumbled upon interesting observation which gave us the impetus for studying the seeding pattern in Assam lemon fruits, which has not been studied across different regions of Assam^4^. Seedlessness is an important character because of the consumer preference for seedless fruits, which offer convenience and culinary applications and Assam lemon has gained popularity over the last few decades because of these desired features^15^. However, recent survey of available market fruits has shown existence of both seeded and non-seeded fruits of Assam lemon, which emphasizes the need to study the seeding pattern among the fruits being grown across Assam. This information can guide *Citrus* growers in making informed decisions regarding the most suitable accessions for Assam lemon cultivation in different regions of Northeast India^16^.

In addition to morphological and seeding variations, the biochemical composition is known to play a crucial role in determining its nutritional value and potential health benefits of Assam lemon^17^. Recent investigations show that Assam lemon is rich several important compounds including citric acid, ascorbic acid, pectin, flavonoids etc. that contribute towards antioxidant properties and other therapeutic attributes^18,19^. However, the concentration of these bioactive compounds may vary among the accessions from different districts of Assam owing to different environmental factors and agricultural practices^20^. Investigating the biochemical variations in Assam lemon across different districts of Assam is expected to provide valuable information for understanding the potential of different populations for applications in functional foods, nutraceuticals, and pharmaceutical industry^21,22^.

Variations in soil composition, including factors such as pH, and the micronutrient and macronutrients quantity may influence the growth, development, and productivity of Assam lemon^23^. By studying the variation in different soil parameters across districts of Assam, a better understanding of soil-Assam lemon interactions can be gained^24^. This knowledge is valuable for optimizing soil management practices, including fertilizer application, irrigation, and soil amendment strategies, to enhance the productivity and quality of Assam lemon^25^.

This study thus aims to provide comprehensive understanding of the morphological variation, soil variation, seeding pattern, and biochemical variation of Assam lemon in different districts of Assam. The findings from this study will benefit *Citrus* growers, breeders, and researchers involved in the cultivation and promotion of Assam lemon in other parts of the globe. 

## Materials and methods

### Study area selection

**Figure 1:**
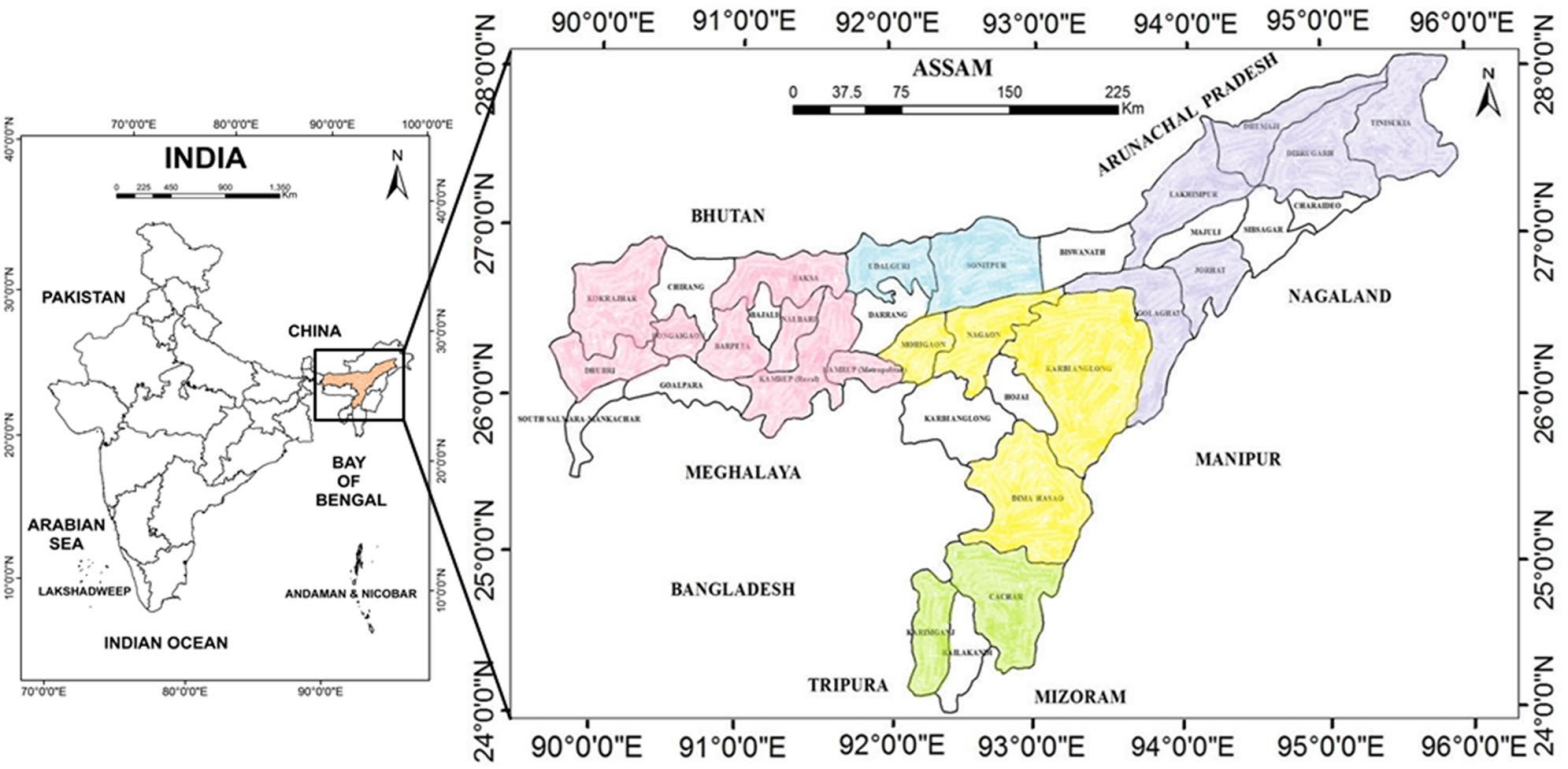
Map of Assam, India showing the study area, prepared using ArcGIS software (version 10.2).

**Figure 2:**
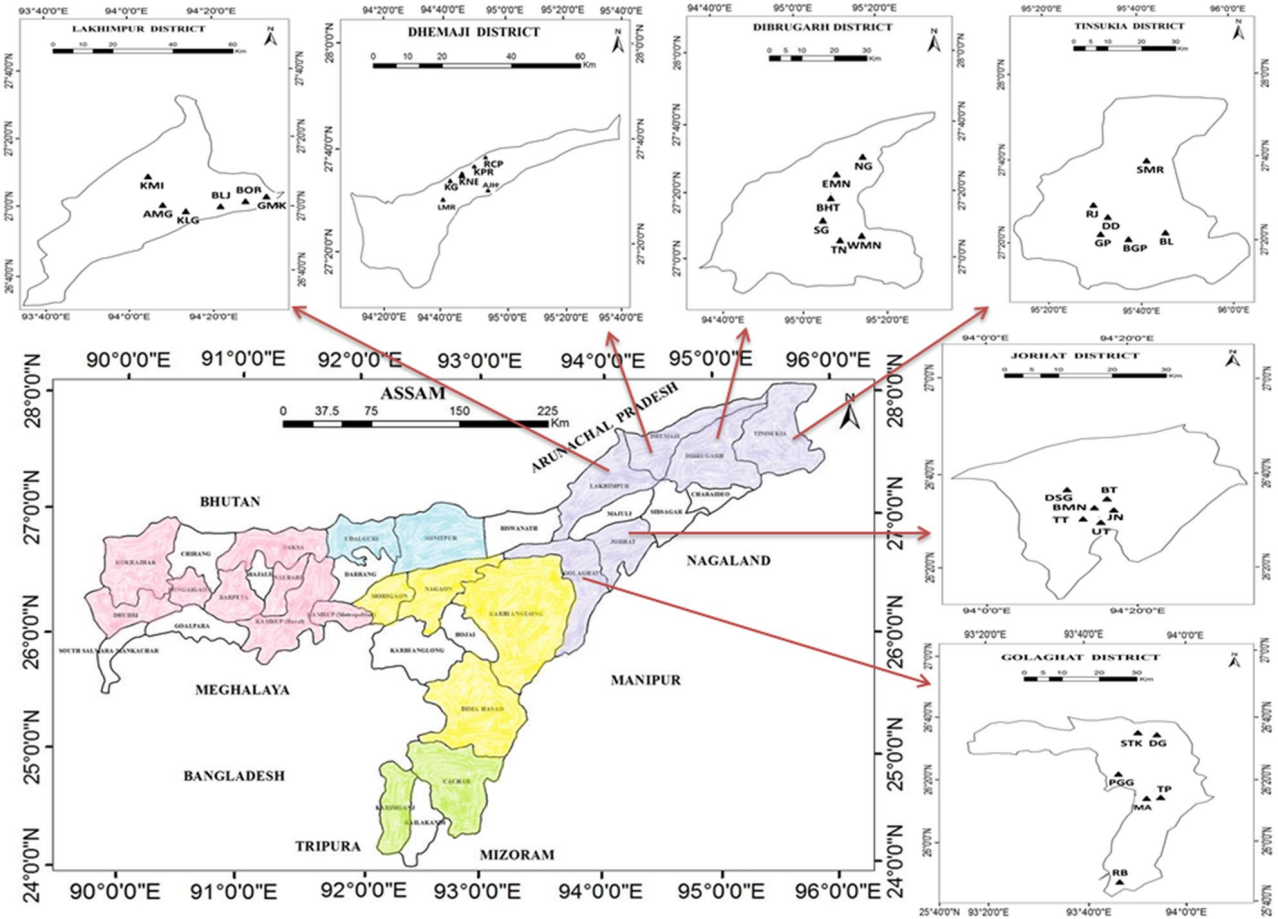
Map of Assam showing the geographic locations of the sampled population in Upper Assam, prepared using ArcGIS software (version 10.2).

**Figure 3:**
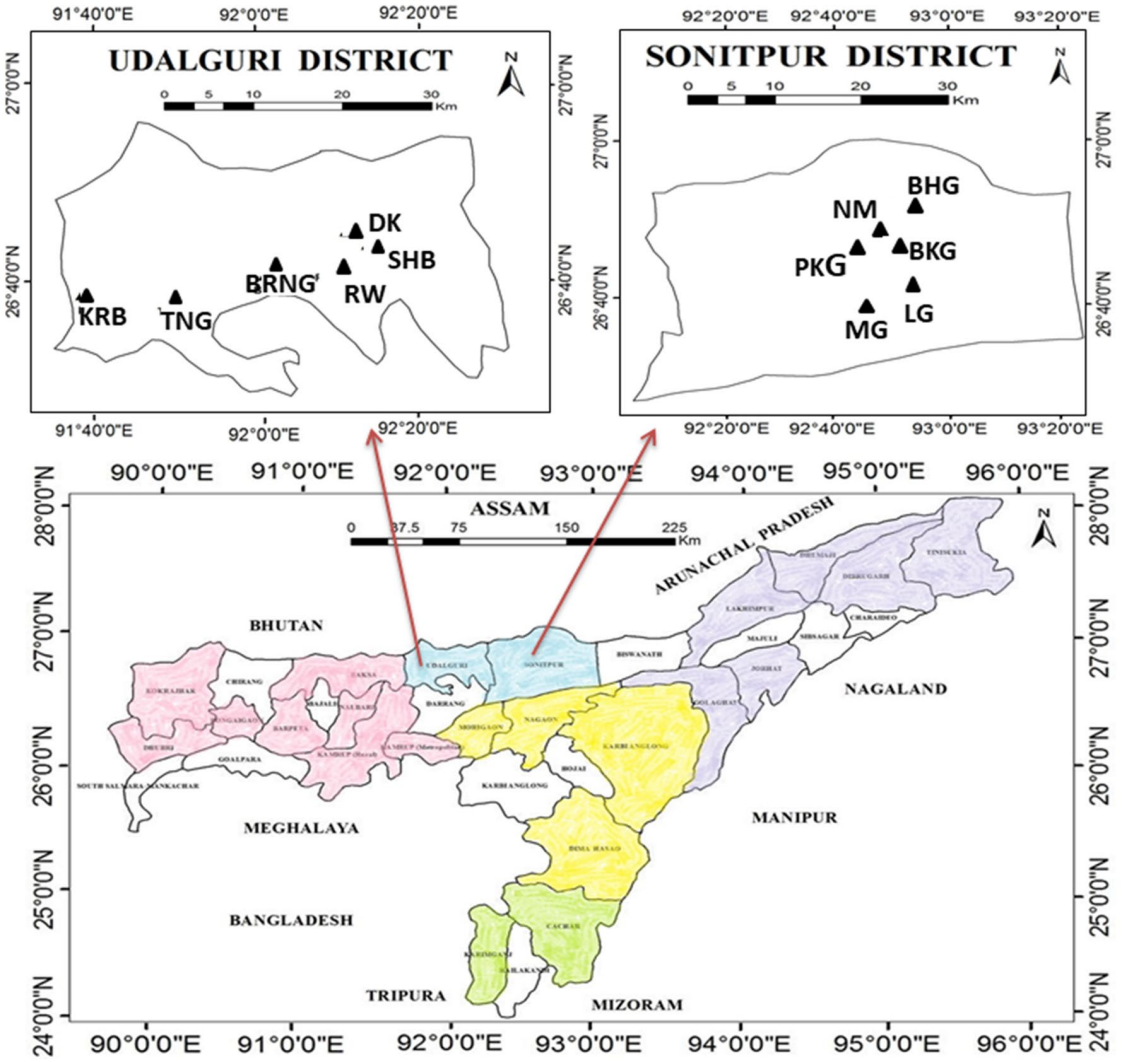
Map of Assam showing the geographic locations of the sampled population in North Assam, prepared using ArcGIS software (version 10.2).

**Figure 4:**
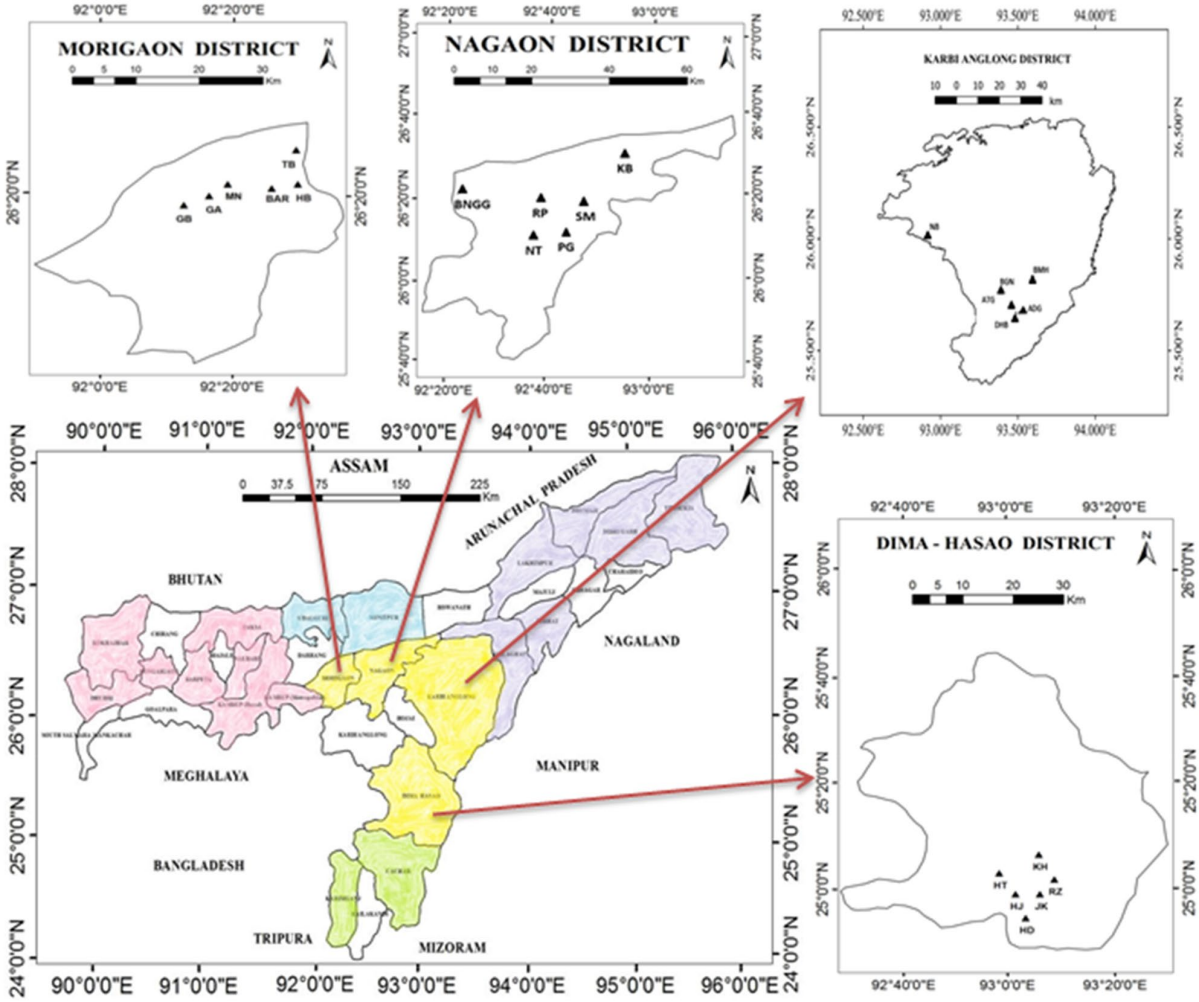
Map of Assam showing the geographic locations of the sampled population in Central Assam, prepared using ArcGIS software (version 10.2).

**Figure 5:**
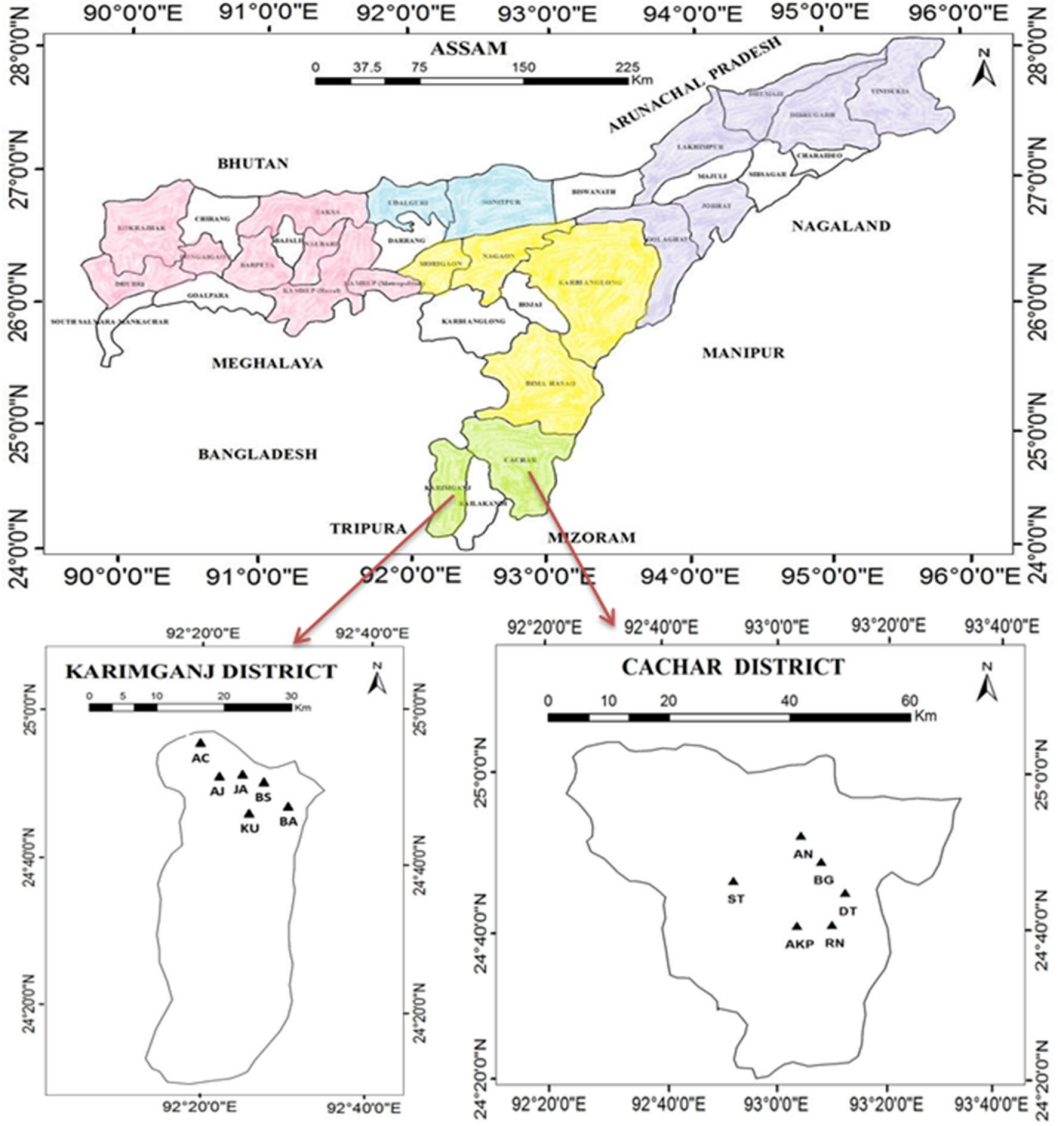
Map of Assam showing the geographic locations of the sampled population in Barak Valley, prepared using ArcGIS software (version 10.2).

**Figure 6:**
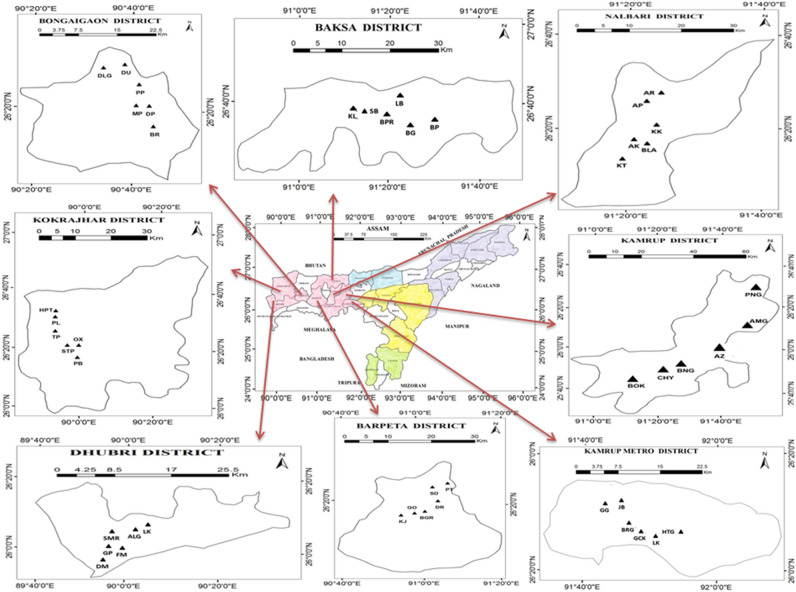
Map of Assam showing the geographic locations of the sampled population in Lower Assam, (prepared using ArcGIS software (version 10.2).

In order to assess the diversity in morphological, seeding pattern, and biochemical characteristics of Assam lemon, a comprehensive study was conducted in 22 districts of Assam, spanning across 5 distinct divisions of the state viz*.* Upper Assam division (Fig. 1, purple hue), North Assam division (Fig. 1, blue hue), Central Assam division (Fig. 1, yellow hue), Barak Valley division (Fig. 1, green hue), and Lower Assam division (Fig. 1, pink hue). The selected districts from Upper Assam include Dhemaji, Dibrugarh, Tinsukia, Lakhimpur, Jorhat, and Golaghat (Fig. 2); the districts from North Assam include Udalguri, and Sonitpur (Fig. 3); from Central Assam, the included districts are Karbi Anglong, Dima Hasao, Nagaon, and Morigaon (Fig. 4); the districts from Barak Valley include Cachar, and Karimganj (Fig. 5); and from Lower Assam, the districts included are Kamrup Metropolitan, Kamrup Rural, Baksha, Nalbari, Barpeta, Bongaigaon, Kokrajhar, and Dhubri (Fig. 6). This careful selection ensured the representation of all regions within Assam for the purposes of this investigation. These districts represent a range of geographical populations, elevation, and soil types prevalent in Assam. A map has been generated using ArcGIS (version 10.2) to illustrate the locations where samples were collected across various districts in Assam. A total of 690 accessions were studied from 132 populations across 22 selected districts in Assam along with the control for morphological variation analysis (Supplementary file 1). The samples were obtained from orchards or homestead garden upon obtaining prior permission from the owner to use the accessions for research. The fruits obtained from the Horticulture Research Station, Kahikuchi, Guwahati under the guidance of Dr. Sarat Saikia were used as control population, owing to them being the progeny of the original stock population.

### Fruit materials

Fresh fruit samples of Assam lemon were systematically collected from a total of 132 populations across 22 selected districts in Assam (Supplementary file 1). Additionally, samples were also obtained from the Horticulture Research Station, Kahikuchi, which served as the control population for comparison (Supplementary file 1). These collected fruit samples were carefully handled and stored in a refrigerated environment at − 80 °C until the completion of the present investigation.

### Morphological analysis

For our research on morphological diversity across the various districts of Assam, we carefully considered a comprehensive set of 70 variables. These variables encompass a wide range of characteristics related to trees, including their physical traits, leaves, flowers, fruits, seeds etc. and the observations were recorded during the time of the investigation. To ensure a thorough analysis, we examined the following aspects which includes, tree character, tree height, branches density, branches angle, thorn density, thorn size, thorn shape, leaf density, leaf division, leaf venation, leaf shape, leaf apex, leaf margin, leaf intensity of green, leaf width, leaf length, flower nature, flower sexuality, flower color, flower length (cm), sepal arrangement, sepal color, sepal length (cm), petal arrangement, petal fusion, petal adaxial side color, petal abaxial side color, petal length (cm), androecium arrangement, androecium length (cm), number of anthers, anther arrangement, anther color, anther length (cm), filament color, filament length (cm), gynoecium arrangement, gynoecium length (cm), sigma color, stigma length (cm), style color, style length (cm), ovary color, ovary length (cm), % flowering shoot, flower density, % full bloom flower, fruit density, fruit color, fruit shape, fruit skin texture, fruit base, fruit apex, fruit axis, fruit weight, fruit length, fruit diameter, specific gravity, number of segments, fruit peel weight, fruit pulp weight, fruit pulp : peel ratio, fruit rind thickness (mm), albedo thickness (mm), flavedo thickness (mm), the length from pith to albedo (mm), pith diameter (mm), seed availability, seed number, seed color.

The data collected was further analyzed using statistical techniques and clustering methods, to identify patterns, relationships, and potentially distinct groups of Assam lemon within the studied regions.

### Seeding pattern

For the investigation of seeding pattern of Assam lemon, mature fruits were sampled from different populations of 22 districts throughout the Assam. In addition, fruits were collected from the Horticulture Research Station, Kahikuchi to serve as a control population for comparison. The mature fruits were carefully collected at their maturity stage (60 DAF) to ensure accurate seed analysis. In the laboratory, the collected fruits were gently washed and dried to remove any external contaminants. Subsequently, each fruit was cut open, and the seeds were carefully extracted using a sterile knife, and forceps. The total number of seeds per fruit was counted, and their color was recorded. The data obtained from the seed analysis were subjected to statistical analysis using methods described below to determine the average number of seeds per fruit and assess any significant variations among different samples (Supplementary file 1).

### Biochemical analysis

The fruit quality characteristics of selected districts of Assam along with control population were assessed, focusing on several parameters (Supplementary file 1). These parameters included pH level, % of juice content, total soluble solids (measured in °Brix), citric acid concentration (measured in g/ml), ascorbic acid concentration (measured in mg/ml), total soluble solids to titratable acidity ratio, total sugar content (measured in µg/ml), reducing sugar content (measured in µg/ml), carotenoid concentration (measured in mg/g), chlorophyll content (measured in µg/g), pectin content (both in the peel and pulp), equivalent weight (for both peel and pulp), methoxy content (for both peel and pulp), anhydrounic acid content (for both peel and pulp), and degree of esterification (for both peel and pulp).

#### pH

Total pH of the Assam lemon juice were measured using pH 700 m (Eutech Instruments, United Kingdom)^26^.

#### Total soluble solids

Total soluble solids of the lemon juice were determined as °Brix using OPTi digital refractometer (Bellingham + Stanley, United Kingdom)^27^.

#### % juice content

% juice content determination was performed following the methodology described by Kashyap et al. in 2020, with slight modifications^28^. The weight of both the fruit and its corresponding juice content were measured in grams and documented. The % of juice content was then calculated using the formula given below,

$$\% \;{\text{Juice}}\;{\text{Content}} = \frac{{{\text{Juice}}\;{\text{Weight}}}}{{{\text{Fruit}}\;{\text{Weight}}}} \times 100$$

#### Citric acid

The determination of citric acid content in the juice of Assam lemon fruits was done using a modified protocol described by Brima et al. in 2014^29^. To measure the concentration of citric acid, the lemon juice was extracted by squeezing the fruits and then diluted with distilled water at a ratio of 1:9 (Juice:Water). Phenolphthalein was added as an indicator, and the titration was conducted using NaOH as the base. The titration process continued until the color of the juice turned red/pink and remained consistent for at least 15 s. This titration was repeated with additional aliquots of the sample solution until concordant results were obtained. The concentration of citric acid was then calculated using the formula given below,

$${\text{Citric}}\;{\text{acid}} = {\text{A}} \times {\text{Acid}}\;{\text{factor}} \times 1$$

where A = Mean value of coordinate readings. Acid factor = 0.0064.

#### Ascorbic acid

The determination of ascorbic acid content in the juice of Assam lemon fruits, a modified protocol described by Satpathy et al. in 2021 was followed^30^. To measure the concentration of ascorbic acid, 20 ml of fresh lemon juice filtrate was transferred into a 250 ml conical flask, and 1 ml of starch indicator was added. The sample solution was then titrated against a 0.01 mol L^−1^ iodine solution until the color changed to a dark black color. The titration process was repeated with additional aliquots of the sample solution until concordant results were obtained. The concentration of ascorbic acid was then calculated using the formula given below,

$${\text{N = CV}}$$

$${\text{N}}_{{{\text{AA}}}} {\text{ = 3N}}$$

$${\text{Ascorbic}}\;{\text{Acid}} = {\text{N}}_{{{\text{AA}}}} \times {\text{M}}_{{{\text{AA}}}}$$

where N = Moles of iodine. C = 0.01 mol L^−1^. V = Mean value of the concordant readings. N_AA_ = Moles of ascorbic acid. M_AA_ = Molecular mass of ascorbic acid.

#### Total soluble solids/titratable acidity (TSS/TA)

The TSS/TA ratio of Assam lemon fruit was assessed using the methodology outlined in the study conducted by Kashyap et al. in 2020^28^. This ratio was obtained by dividing the °Brix value (representing the Total Soluble Solids) by the percentage of acid content.

#### Total sugar and reducing sugar

The quantification of reducing sugar was performed using 3,5-dinitro salicylic acid (DNS), following the modified protocol described by Gusakov et al. in 2011^31^. Additionally, the determination of total sugar was conducted using Anthrone reagent, as outlined by Buckan in 2015 with slight modifications^32^.

#### Carotenoid content

The carotenoid content of the peel samples from Assam lemon was determined using the methodology described by Kashyap et al. in 2020, with slight modifications^28^. 1 g sample of fruit was ground with liquid nitrogen, and the resulting powder was mixed with 10 ml of a hexane:acetone:ethanol solution (v/v; 50:25:25). Subsequently, the mixture was centrifuged at 4000 g for 5 min. The supernatant, containing the color compounds, was collected, and the volume was adjusted to 10 ml with the extraction solvent. The total carotenoid content was then measured by assessing the absorbance at 450 nm using a spectrophotometer. The concentration of carotenoids was calculated using the following equation,

$${\text{Carotenoid}}\;{\text{Content}} = \frac{{{\text{A}}_{450} \times {\text{ml}}\; {\text{of}}\; {\text{n-hexane}} \times 1.11 \times 100 \times {\text{dilution}}}}{{2500 \times g\; {\text{sample}}}}$$

where A = Absorbance at 450 nm wavelength. V = Total volume of sample. W = Weight of fresh plant tissue.

#### Chlorophyll content

The chlorophyll content of the juice of Assam lemon at various fruit developmental stages were determined using the protocol described by Kashyap et al. 2020 with minor modification^28^. For the determination of chlorophyll concentration in the fruit peel, 1 g of peel sample was ground with liquid nitrogen. The resulting powder was then mixed with 20 ml of 80% acetone solution containing 0.5 g of MgCO_3_. After incubating the mixture at 4 °C for 3 h, it was centrifuged at 2500 rpm for 5 min. The supernatant was carefully collected, and its absorbance was measured at 654 nm and 663 nm using a spectrophotometer. Using the provided equation, the concentration of chlorophyll was calculated.

$${\text{Chlorophyll}}\;{\text{a}} = 12.7 \left( {{\text{A}}_{663} } \right) - 2.69 \left( {{\text{A}}_{645} } \right)$$

$${\text{Chlorophyll b = 22}}{.9 }\left( {{\text{A}}_{{{645}}} } \right) - { 4}{\text{.68 (A}}_{{{663}}} {)}$$

$${\text{Total}}\;{\text{Chlorophyll = 20}}{.2 }\left( {{\text{A}}_{{{645}}} } \right){ + 8}{\text{.02 (A}}_{{{663}}} {)}$$

where A = Absorbance at specific wavelength. V = Final volume of chlorophyll extract in 80% acetone. W = Fresh weight of tissue extracted.

#### Pectin content

The determination of pectin content in both the peel and pulp samples of Assam lemon was done following the protocol outlined by Khamsucharit et al. in 2017, with minor modifications^33^. A total of 5 g of dried fruit sample was mixed with 100 ml of Citric acid solution (prepared by mixing 9 parts distilled water with 1 part Citric acid) and incubated at 65 °C for 1 h. After the completion of incubation, the extract was filtered, and 95% ethanol was added to the filtrate in a 1:1 proportion. The mixture was then incubated at room temperature for 2 h. Following incubation, the flocculants were skimmed off and washed 2–3 times using ethyl alcohol. The resulting precipitate was thoroughly dried at 35–40 °C and weighed to determine the pectin concentration. The concentration of pectin was calculated using the provided equation.

$${\text{Ypec }}\left( \% \right) = \frac{{\text{P}}}{{{\text{Bi}}}} \times 100$$

where Ypec = Yield of pectin. P = Amount of extracted pectin. Bi = Initial amount of fruit powder.

#### Equivalent weight

The determination of equivalent weight content in both the peel and pulp samples of Assam lemon was done following the protocol outlined by Khamsucharit et al. in 2017, with minor modifications^33^. In this process, 0.5 g of dried pectin sample obtained from the previous pectin estimation experiment was dissolved in 5 ml of ethanol. To this pectin solution, 1 g of NaCl and a few drops of phenol red indicator were added. The resulting mixture was titrated against 0.1 M NaOH until a pale pink color, indicating the end point, was achieved. The titration was repeated with additional aliquots of the sample solution until consistent and concordant results were obtained. The equivalent weight was then calculated using the provided equation.

$${\text{Equivalent}} \;{\text{Weight}} = \frac{{{\text{Weight}} \;{\text{of}} \;{\text{pectin}}\; {\text{samples}}\; \times \; {\text{Molarity}}\; {\text{of}}\; {\text{alkali}}}}{{{\text{Volume}}\; {\text{of}}\; {\text{alkali}}}} \times 100$$

#### Methoxy content (MeO)

The determination of methoxy content in both the peel and pulp samples of Assam lemon was done following the protocol outlined by Khamsucharit et al. in 2017, with minor modifications^33^. The methoxy content was estimated using the neutralized titrated solution obtained from the equivalent weight estimation. To the solution, 0.25 M NaOH was added and stirred for 30 min, followed by the addition of 25 ml of 0.25 N HCl. The resulting mixture was titrated against 0.1 N NaOH until a pale pink color, indicating the end point, was achieved. The titration was repeated with additional aliquots of the sample solution until consistent and concordant results were obtained. The methoxy content was then calculated using the provided equation.

$${\text{MeO }}(\% ) = \frac{{{\text{Volume}}\; {\text{of}}\; {\text{alkali}} \times {\text{Normality}} \; {\text{of}}\; {\text{alkali}} \times 31}}{{{\text{W}} \times 1000}} \times 100$$

#### Anhydrounic acid (AUA)

The anhydrounic acid content in both the peel and pulp samples of Assam lemon was determined according to the method described by Khamsucharit et al. in 2017, with slight modifications^33^. The calculation of anhydrounic acid content was performed using the provided equation.

$${\text{AUA}} \left( \% \right) = \frac{{176 \times 0.1 \times {\text{Z}} \times 100}}{{{\text{W}} \times 1000}} + \frac{{176 \times 0.1 \times {\text{Y}} \times 100}}{{{\text{W}} \times 1000}}$$

where Molecular weight = 176 g. Z = Equivalent weight titration result. Y = Methoxy content titration result. W = Weight of sample.

#### Degree of esterification (DE)

The degree of esterification in both the peel and pulp samples of Assam lemon was determined using the method described by Khamsucharit et al. in 2017, with slight modifications^33^. The calculation of the degree of esterification was carried out using the equation provided.

$${\text{DE}} = \frac{{176 \times {\text{MeO}} (\% ) \times 100}}{{31 \times {\text{AUA}} (\% )}}$$

where DE > 50% = High methoxy pectins (HM). DE < 50% = Low methoxy pectins (LM).

### Statistical analysis

For statistical analysis, the numerical data was generated using morphological characters including seeding pattern and also biochemical attributes which was used for the statistical analysis during the current investigation^34,35^. Further, using the PAST 4.11 program, we performed, Principal Coordinate Analysis (PCoA), and constructed a dendrogram using the Unweighted Pair Group Method with Arithmetic Mean (UPGMA) based on these numerical data generated using morphological characters. Also, using the PAST 4.11 program, Analysis of Variance (ANOVA) test with post hoc Duncan Multiple Range Test (DMRT) was performed using SPSS 26 program to determine significant differences in morphological and biochemical attributes.

### Soil nutrient estimation

Soil samples were carefully collected from various districts of Assam, focusing on locations with thriving Assam lemon trees during their active growth phase. Care was taken to avoid areas with apparent disturbances. Soil samples were collected to a depth of 15 cm, and several sub-samples were combined to create a composite sample at each site^36^. The collected soil samples were preserved and transported to the laboratory for analysis, where various physicochemical properties, including, pH, availability of macronutrients and micronutrients were measured. To assess the soil conditions in the 22 districts of Assam along with the control population, we employed soil testing kits (K054-1KT and K095L-1KT, Himedia) (Supplementary file 1). These kits enabled us to estimate the concentrations of both micronutrients and macronutrients present in the soil samples collected for the current investigation. In terms of micronutrients, we focused on assessing the levels of Copper, Zinc, Boron, Manganese, Iron, and Molybdenum. These elements play crucial roles in plant growth and development, and their availability in the soil can significantly impact the health and productivity of vegetation. For macronutrient analysis, we evaluated several key parameters, including pH levels, organic Carbon content, Phosphate concentration, Potassium levels, Ammoniacal Nitrogen content, and Nitrate Nitrogen levels. These macronutrients are essential for plant nutrition, as they are involved in various physiological processes and influence overall soil fertility.

This study complied with relevant institutional, national, and international guidelines and legislation of India, and no specific permits were required to collect the plant materials. The authentication of the collected samples was done by Department of Botany, Gauhati University under the guidance of curator Dr. Souravjyoti Borah. The voucher specimen of the collected plant samples has been deposited in the herbarium of Department of Botany at Gauhati University and the accession number GUBH19963 was issued for the same.

## Results

### Morphological analysis

**Figure 7:**
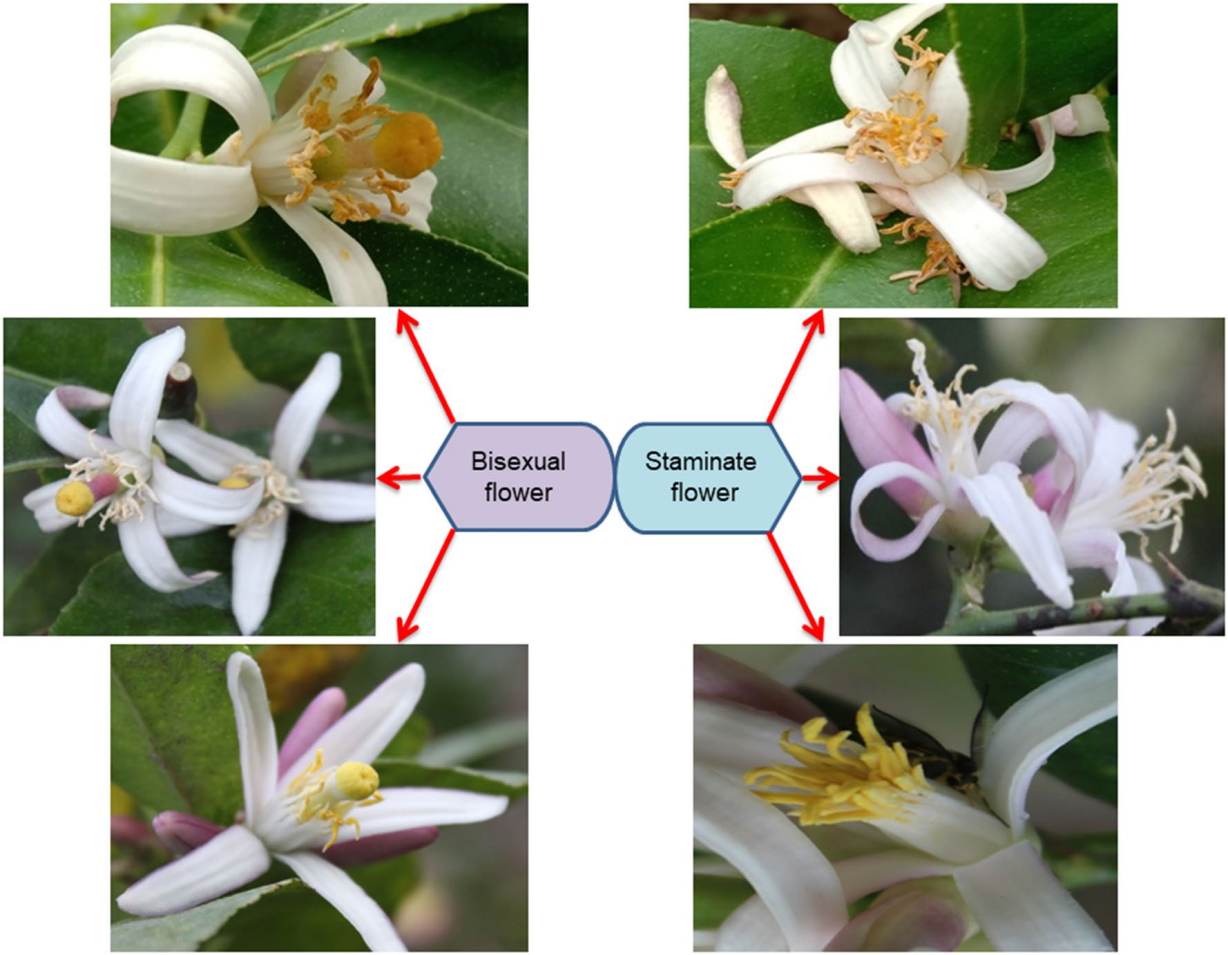
Floral morphology of Assam lemon: Bisexual flower (left); Staminate flower (right).

**Figure 8:**
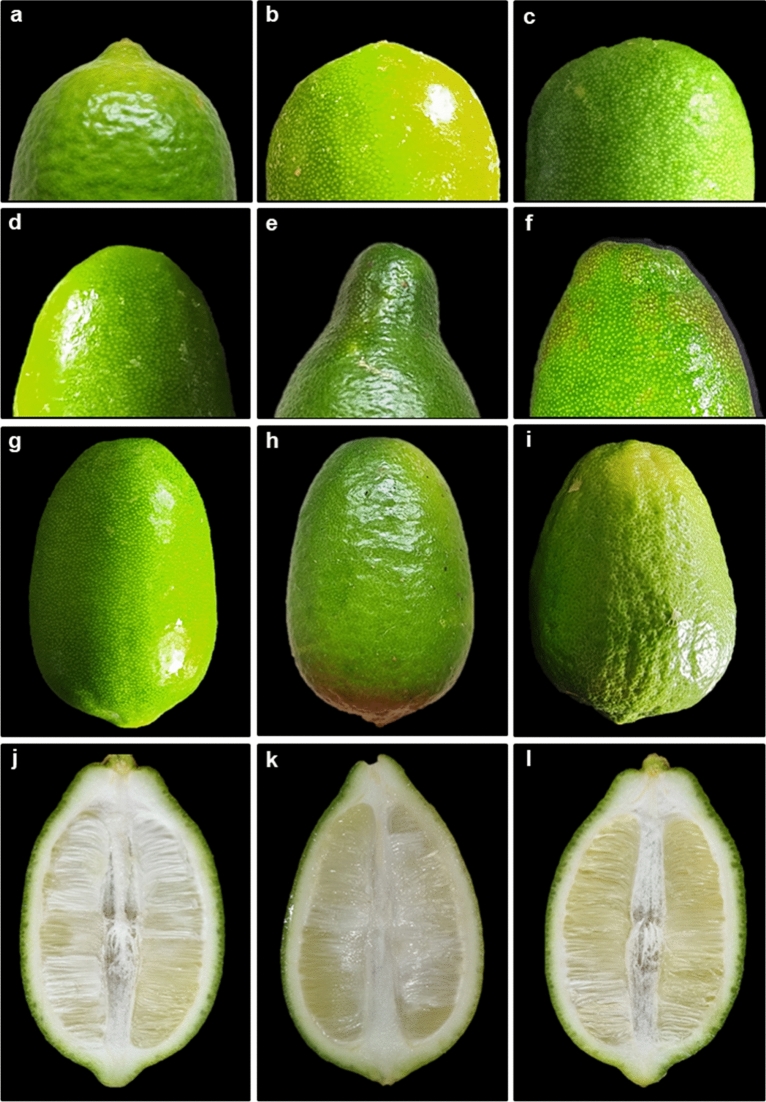
Fruit attributes of Assam lemon; (**a–c**)**:** Shape of the base of the fruit (**a**)**:** mammiform, (**b**)**:** acute, (**c**)**:** round), (**d–f**)**:** shape of the apex of the fruit (**d**)**:** round, (**e**)**:** necked, (**f**)**:** truncate), (**g–i**)**:** texture of the fruit (**g**)**:** smooth, (**h**)**:** rough, (**i**)**:** bumpy), (**j-l**)**:** fruit axis (**j**)**:** semi-hollow, (**k**)**:** solid, (**l**)**:** hollow).

The analysis of morphological characters in Assam lemon revealed significant variation across different regions of the state (Supplementary file 2, Supplementary file 3, Supplementary file 4). The study encompassed a total of 70 morphological characters, including tree characteristics, thorn characters, leaf traits, flower attributes, fruit properties, and seed features. Among all the studied characters, the flower traits exhibited the most pronounced variation. Notably, the investigation of Assam lemon flowers indicated that accessions from control, Dhemaji, Tinsukia, Jorhat, and Dibrugarh displayed both bisexual and unisexual flowers but, the concentration of unisexual flowers was comparatively quite less in these regions. In contrast, accessions from Golaghat, Central Assam (Karbi Anglong, Dima Hasao, Nagaon, and Morigaon), North Assam (Udalguri, and Sonitpur), Lower Assam (Kamrup Metropolitan, Kamrup Rural, Baksha, Nalbari, Barpeta, Bongaigaon, Kokrajhar, and Dhubri) to Barak valley (Dima Hasao, Cachar, and Karimganj) exhibited a combination of bisexual and unisexual flowers, having unisexual flowers of almost equal concentration to that of bisexual flowers. The unisexual flowers possessed only the androecium (Fig. 7). Furthermore, the study revealed a distinct difference in the number of anthers between unisexual and bisexual flowers. Unisexual flowers were found to have as much as 40 anthers, whereas bisexual flowers exhibited 36 anthers (Supplementary file 3). Additionally, significant variations in fruit morphological characters were also observed across 690 accessions of Assam (Fig. 8, Supplementary file 4).

### Seeding pattern

**Figure 9:**
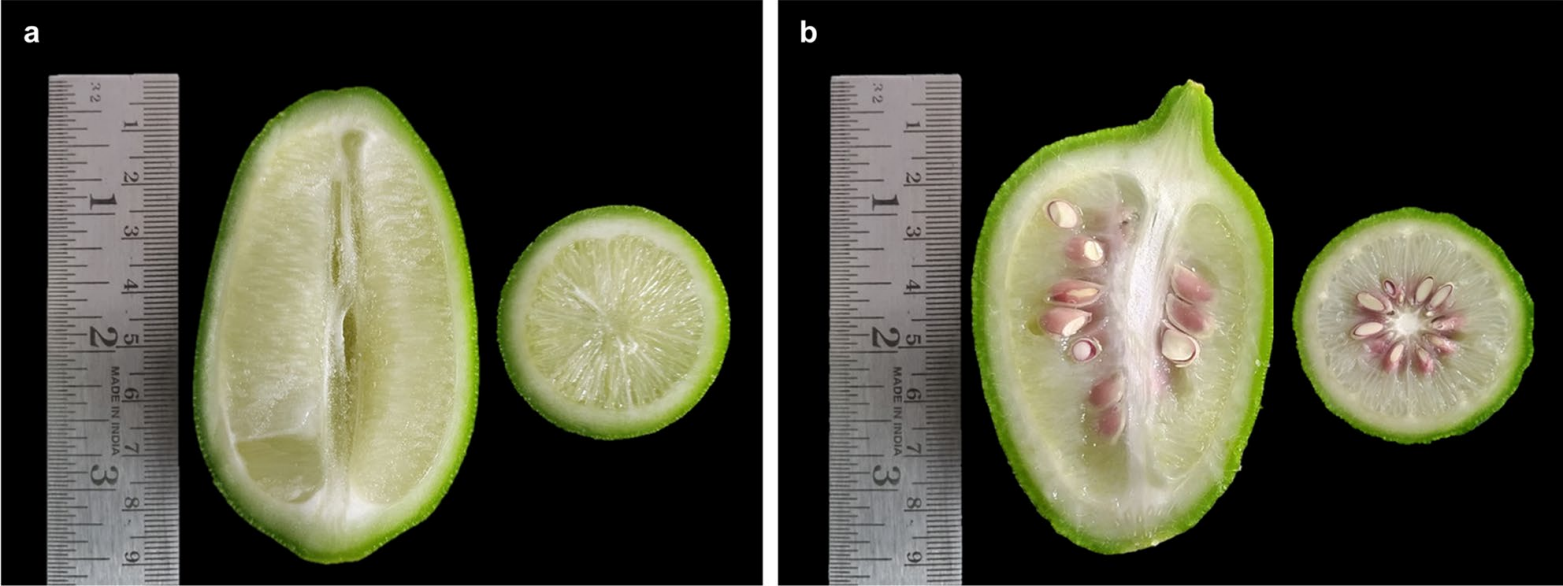
Assam lemon fruit; (**a**)**:** Seedless fruit, (**b**)**:** Seeded fruit.

**Figure 10:**
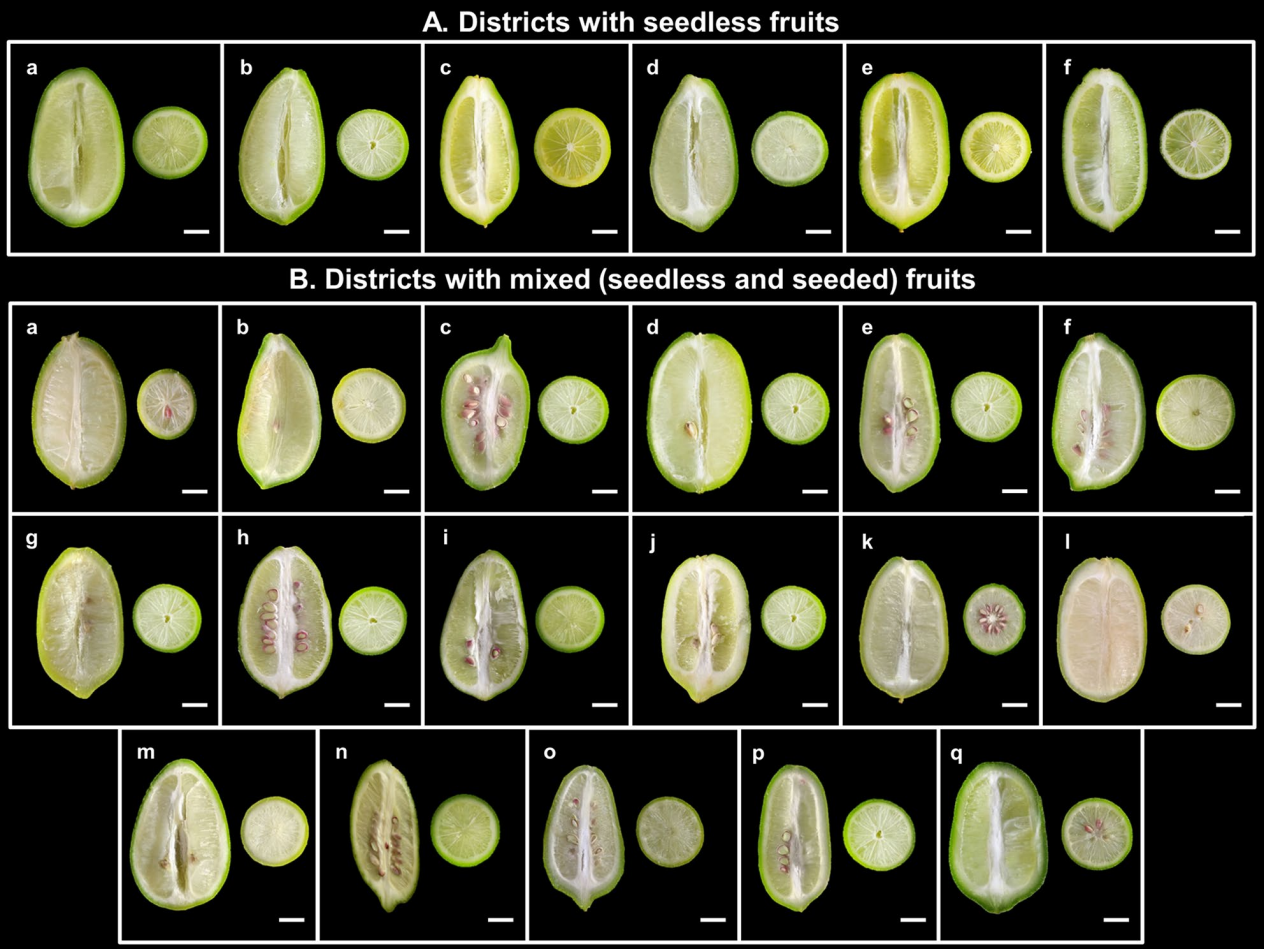
Variation in seed availability in Assam lemon fruits across different districts of Assam; **A:** Districts with seedless fruit) (**a):** Control, (**b):** Tinsukia, (**c):** Dhemaji, (**d):** Lakhimpur, (**e**)**:** Dibrugarh, (**f**)**:** Jorhat), (**B**)**:** Districts with mixed (Seedless and Seeded) fruits (**a):** Golaghat, (**b):** Sonitpur, (**c**)**:** Karbi Anglong, (**d**)**:** Nagaon, (**e**)**:** Morigaon, (**f**)**:** Udalguri, (**g**)**:** Baksha, (**h):** Nalbari, (**i**)**:** Barpeta, (**j**)**:** Kamrup Metro, (**k**)**:** Kamrup Rural, (**l**)**:** Bongaigaon, (**m**)**:** Kokrajhar, (**n**)**:** Dhubri, (**o**)**:** Dima Hasao, (**p**)**:** Cachar, (**q**)**:** Karimganj), Scale Bar: 1.5 cm.

During the investigation into the seeding pattern of Assam lemon across the 22 districts of Assam, an interesting finding emerged. It was observed that there is a combination of seeding patterns within the studied districts, with some districts exhibiting the true seedless trait characteristic of Assam lemon, while others exhibited a mixed character and had both seedless and seeded type (Fig. 9). Also, we have observed that in the study area of Upper Assam districts and control, the Assam lemon trees were cultivated as a standalone crop. However, in other selected districts, the Assam lemon trees were found to be cultivated as standalone or alongside other *Citrus* varieties. Throughout the current investigation, we observed that the fruits collected from Tinsukia, Dhemaji, Lakhimpur, Dibrugarh, and Jorhat districts including control population were seedless. However, during the current investigation, we found a mixture of seedless and seeded fruits in the populations of Golaghat district, districts of Central Assam, North Assam, Lower Assam and Barak Valley districts. Interestingly, it appears that Golaghat district serves as the linking district where both the seeded and seedless fruits are present connecting the regions of Central Assam, North Assam, Lower Assam and Barak valley (Fig. 10). Surprisingly, we discovered significant variation in the presence and quantity of seeds among the different districts of Central Assam, North Assam, Lower Assam and Barak Valley. Each district exhibited its own distinct seed count in the fruits where seeds were observed. While, studying the seed count of Assam lemon fruits, we made some interesting observations accessions from Golaghat, Sonitpur, Nagaon, Morigaon, and Nalbari districts consistently exhibited a similar pattern, with seed counts ranging from 0 to 5 seeds per district. On the other hand, when studying the accessions from Karbi Anglong, Udalguri, Baksha, Kamrup Metro, and Kamrup Rural districts, we found the seed counts ranged from 0 to 10 seeds per districts. Additionally, accessions from Barpeta, Bongaigaon, Kokrajhar, Dhubri, Dima Hasao, Cachar, and Karimganj districts also showed mixed seed counts, varying from 0 to > 10 seeds per accession, however, certain accessions from the Dhubri and Cachar regions exhibited a notably high seed count, surpassing 20 seeds per accession (Supplementary file 4).

### PCoA analysis

**Figure 11:**
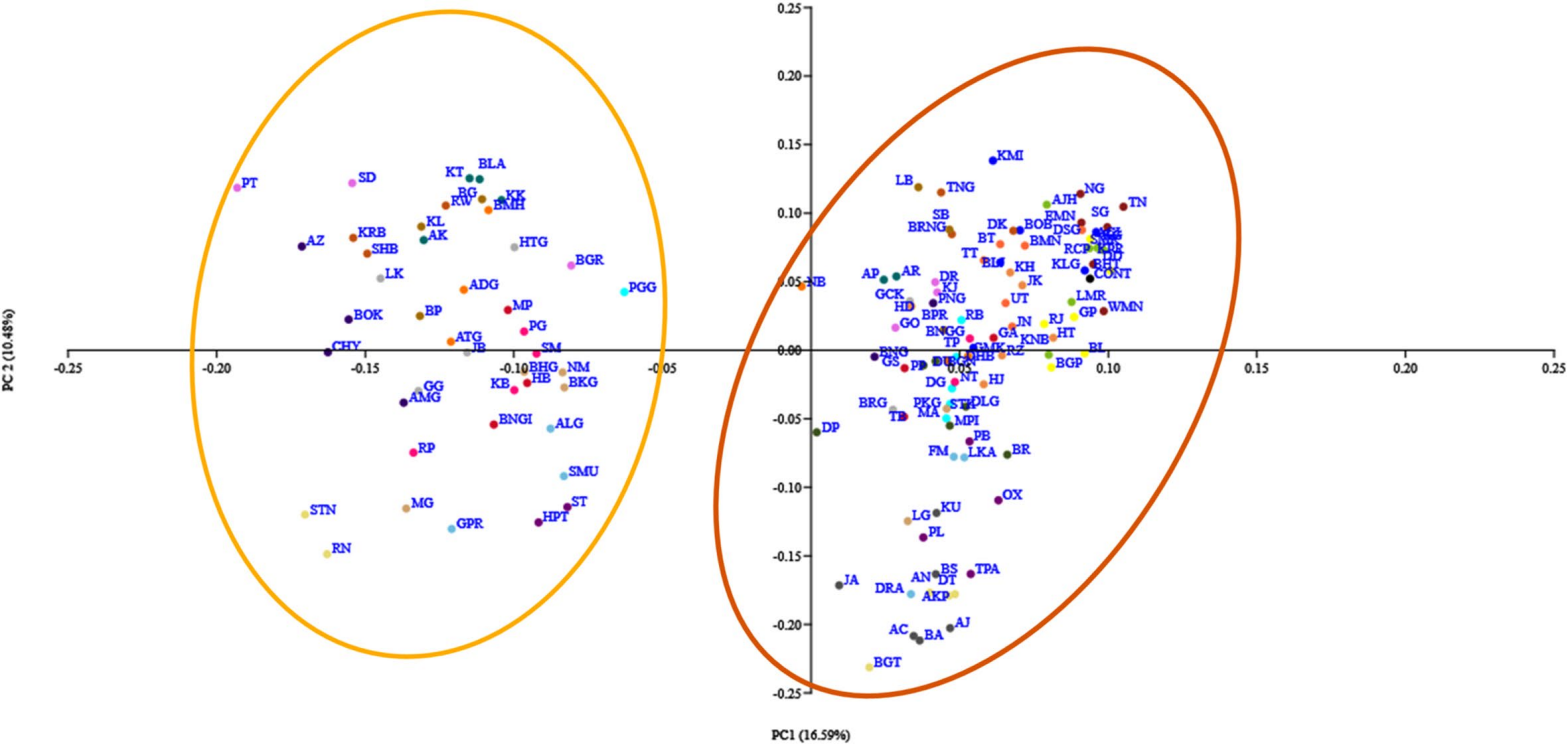
Principal Coordinate Analysis of Assam lemon using morphological characters.

A Principal Coordinates Analysis (PCoA) was conducted to visualize the spatial patterns of morphological variation among populations and districts of Assam lemon. The results of the analysis showed that the populations from districts of Dhemaji, Tinsukia, Dibrugarh, Lakhimpur, Jorhat, and Dima Hasao shared highest morphological similarities with the control population (Fig. 11). On the other hand, the populations from the districts of, Kamrup Metro, Kamrup Rural, Nalbari, Barpeta, Sonitpur, Nagaon, Morigaon, Baksha, Udalguri, Bongaigaon, Dhubri, Kokrajhar, Karbi Anglong, and Cachar exhibited greater dissimilarity from the control population in terms of morphological characteristics. Interestingly, the Ponkagaon (PGG) population of Golaghat district exhibited significant dissimilarity from the control population, while other Golaghat district populations maintained close similarities.

### Cluster analysis

**Figure 12:**
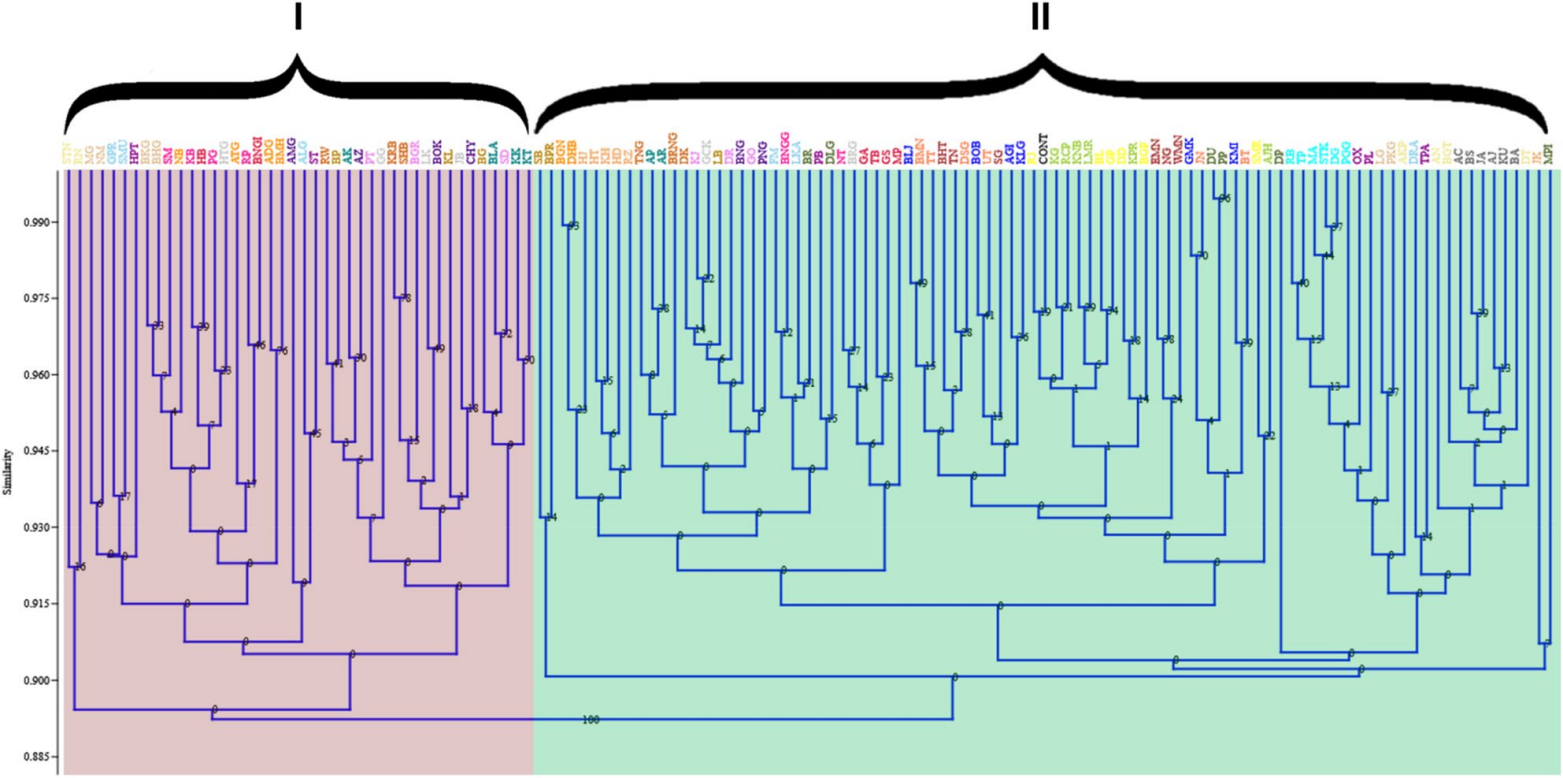
UPGMA analysis of Assam lemon populations using morphological characters. The alphabets bottom represents different populations code and their representative districts selected during the current study; **Control** (CONT), **Tinsukia** (BL, BGP, DD, GP, RJ, SMR), **Dhemaji** (KNB, LMR, KG, KPR, AJH, RCP), **Lakhimpur** (BLJ, BOB, GMK, AHI, KLG, KMI), **Dibrugarh** (EMN, NG, BHT, SG, TN, WMN), **Jorhat** (BMN, TT, JN, UT, DSG, BT), **Golaghat** (RB, PGG, MA, TP, STK, DG), **Sonitpur** (MG, LG, BKG, PKG, NM, BHG), **Karbi Anglong** (NB, ATG, ADG, BMH, BGN, DHB), **Nagaon** (RP, KB, BNGG, NT, PG, SM), **Morigaon** (BNGI, TB, HB, MP, GS, GA), **Udalguri** (RW, TNG, BRNG, KRB, SHB, DK), **Baksha** (BP, KL, SB, BG, BPR, LB), **Nalbari** (AK, BLA, KK, KT, AP, AR), **Barpeta** (PT, SD, DR, BGR, GO, KJ), **Kamrup Metro** (GG, JB, LK, BRG, HTG, GCK), **Kamrup Rural** (AZ, CHY, BOK, AMG, PNG, BNG), **Bongaigaon** (DU, PP, MPI, DP, DLG, BR), **Kokrajhar** (PL, TPA, HPT, ST, OX, PB), **Dhubri** (GPR, FM, DRA, ALG, LKA, SMU), **Dima Hasao** (HT, HJ, HD, KH, JK, RZ), **Cachar** (AN, BGT, STN, DT, RN, AKP), **Karimganj** (AC, AJ, BA, BS, JA, KU).

The dendrogram-based UPGMA algorithm analysis of morphological and seeding pattern data at the district level, led to identification of two major clusters in the resulting dendrogram. From the dendrogram based on morphological data, it was observed that the populations from Tinsukia and Dhemaji exhibited the close clustering with the control population (Fig. 12).

### Biochemical analysis

The analysis of biochemical parameters in Assam lemon samples collected from various districts of Assam revealed substantial variation across the studied regions. Interestingly, we also observed a striking similarity between the biochemical parameters of the control populations and those from the districts of Dhemaji, Tinsukia, Lakhimpur, Dibrugarh, and Jorhat.

#### pH

**Figure 13:**
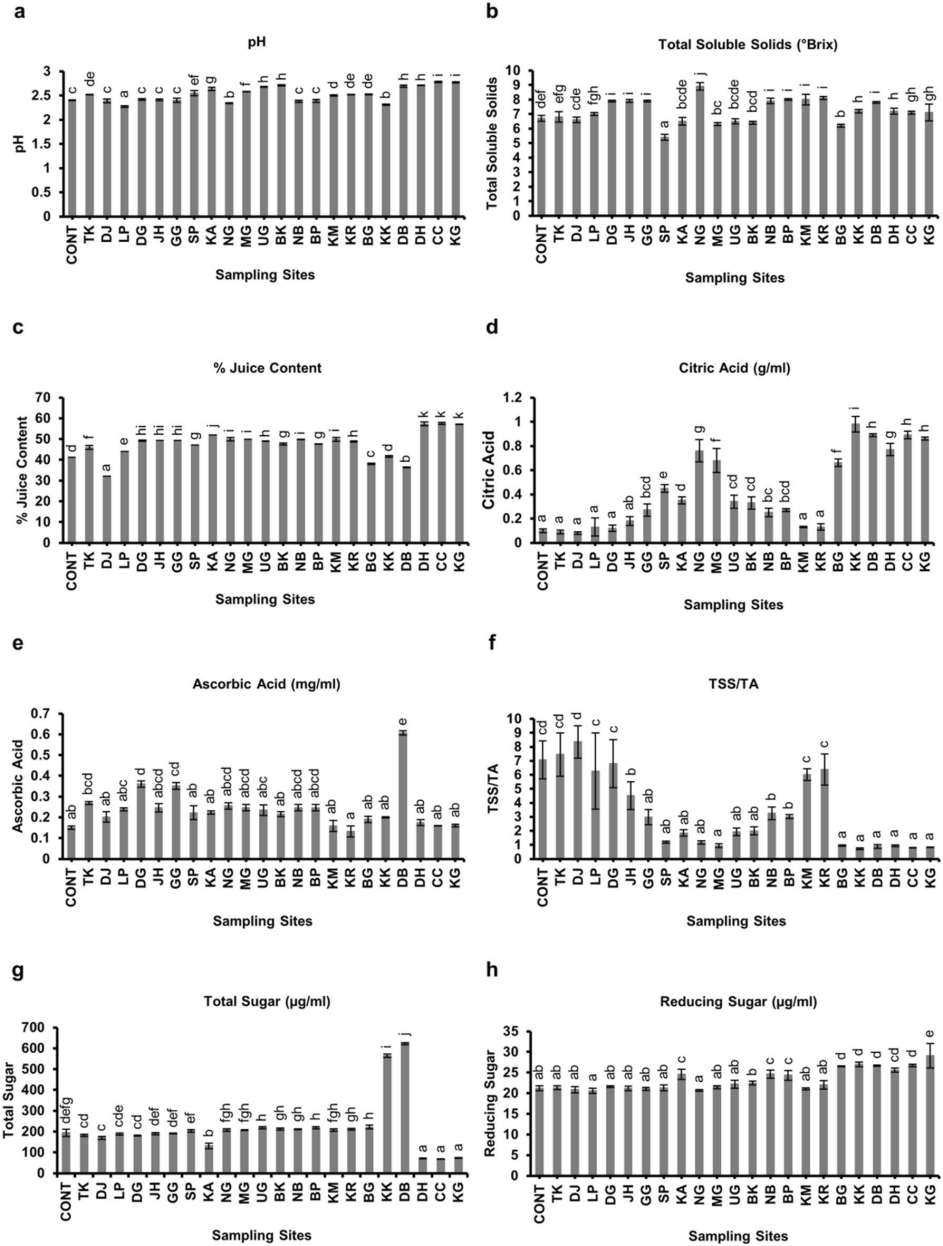
Biochemical characteristic of Assam lemon fruit juice across different districts of Assam; (**a**)**:** pH, (**b**)**:** TSS, (**c**)**:** % Juice Content, (**d**)**:** Citric Acid, (**e**)**:** Ascorbic Acid, (**f**)**:** TSS/TA, (**g**)**:** Total Sugar, (**h**)**:** Reducing Sugar. Data represent mean values (column bars) with standard deviation of n = 3 biological replicates represented as vertical lines on the column bars. Different letters on top of the bars represent significant differences between samples at subset for alpha = 0.05, based on Duncan Multiple Range Test (DMRT) test.

The results of the pH analysis of different districts in Assam along with the control population revealed that samples from the Cachar district had the highest pH (2.78 ± 0.01), while the samples from Lakhimpur district showed the lowest (2.27 ± 0.02) (Fig. 13, Supplementary file 5).

#### % Juice content

The % juice content analysis of different districts in Assam and the control population showed significant variations in the juice content of the samples. The samples from Cachar district had the highest % juice content (57.61 ± 0.523%), while, the samples from Dhemaji district exhibited the lowest % juice content (32.13 ± 0.036%) (Fig. 13, Supplementary file 5).

#### Total soluble solids

The results of the total soluble solids content analysis for different districts in Assam along with the control population revealed that the samples from Nagaon district had the highest total soluble solids (8.9 ± 0.27°Brix), while Sonitpur had the lowest (5.4 ± 0.2°Brix) (Fig. 13, Supplementary file 5).

#### Citric acid

The results of the citric acid content analysis for different districts in Assam along with the control population revealed that Kokrajhar had the highest citric acid content (0.98 ± 0.064 g/ml), while Dhemaji had the lowest (0.08 ± 0.01 g/ml) (Fig. 13, Supplementary file 5).

#### Ascorbic acid

The results of the ascorbic acid content analysis for different districts in Assam along with the control population revealed that Dhubri had the highest ascorbic acid content (0.607 ± 0.011 mg/ml), while Kamrup Rural had the lowest (0.132 ± 0.026 mg/ml) (Fig. 13, Supplementary file 5).

#### Total sugar and reducing sugar

The results of the total sugar content esitimation for different districts in Assam, as well as the control population, revealed that Dhubri had the highest concentration of total sugar content (622.38 ± 5.646 µg/ml) and Cachar had the lowest (67.67 ± 0.97 µg/ml). Furthermore, results of the reducing sugar content analysis showed that Kokrajhar had the highest concentration of reducing sugar content (26.99 ± 0.6 µg/ml), while the lowest concentration was found in Lakhimpur (20.56 ± 0.6 µg/ml) (Fig. 13, Supplementary file 5).

#### Carotenoid content

**Figure 14:**
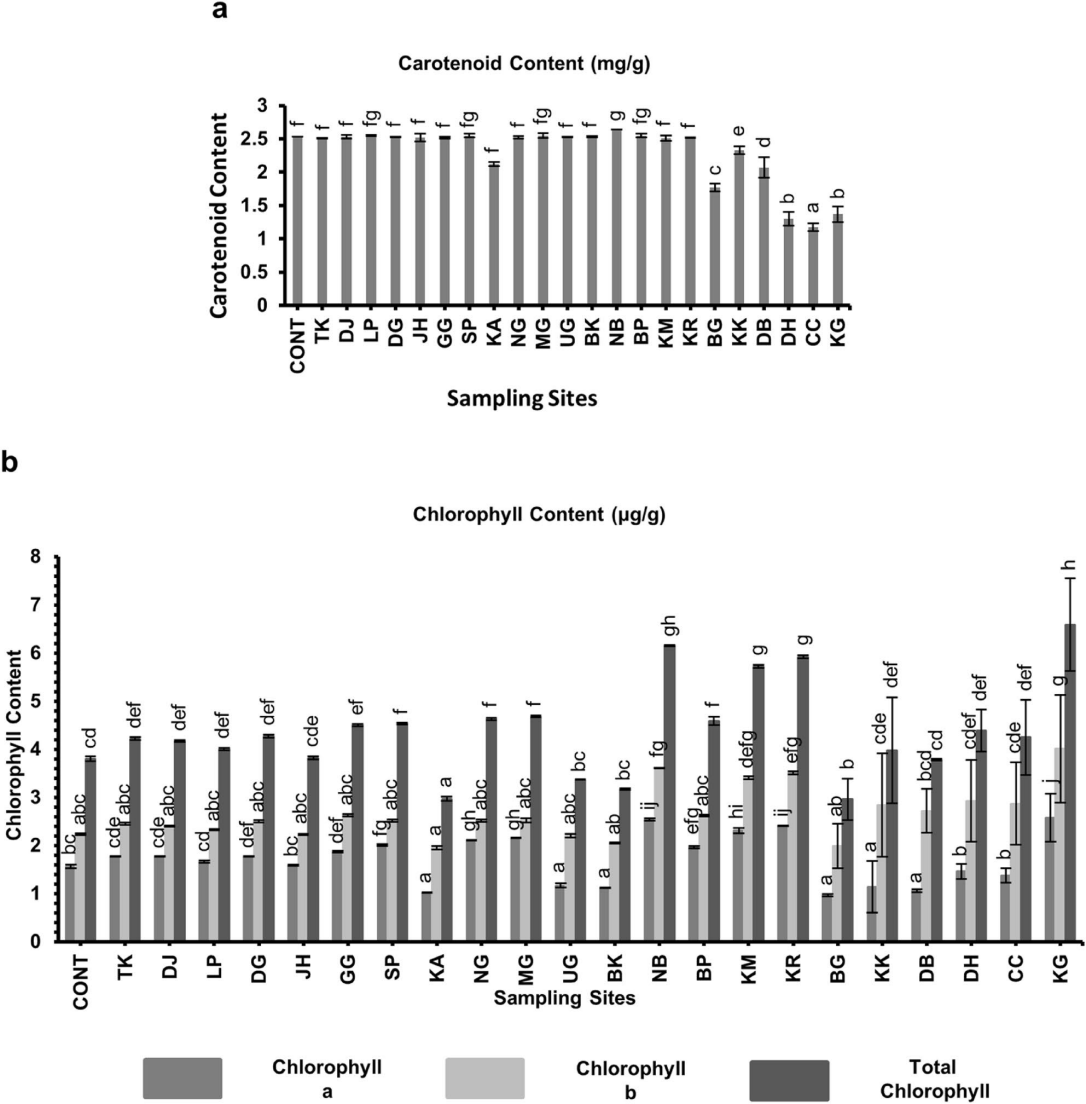
Biochemical characteristic of Assam lemon fruit juice across different districts of Assam; (**a**)**:** Carotenoid Content, (**b**)**:** Chlorophyll Content. Data represent mean values (column bars) with standard deviation of n = 3 biological replicates represented as vertical lines on the column bars. Different letters on top of the bars represent significant differences between samples at subset for alpha = 0.05, based on Duncan Multiple Range Test (DMRT) test.

The results of the carotenoid estimation for the various districts in Assam along with the control population showed that the higest concentration of carotenoid on the peel of Assam lemon fruit was observed in the Nalbari district (2.64 ± 0.001 mg/g), while the lowest concentration was observed in Cachar district (1.17 ± 0.0058 mg/g) (Fig. 14, Supplementary file 5).

#### Chlorophyll content

The results of the chlorophyll content estimation for the various districts in Assam, as well as the control population showed that the highest concentration of Chlorophyll a, Chlorophyll b, and the total chlorophyll content on the peel of Assam lemon fruit was observed in Karimganj district (2.58 ± 0.0496 µg/g, 4.01 ± 0.012 µg/g, 6.59 ± 0.0958 µg/g), and the lowest concentration was observed in Bongaigaon district (0.97 ± 0.0027 µg/g, 1.99 ± 0.046 µg/g, 2.96 ± 0.0434 µg/g) respectively (Fig. 14, Supplementary file 5).

#### Pectin content

**Figure 15:**
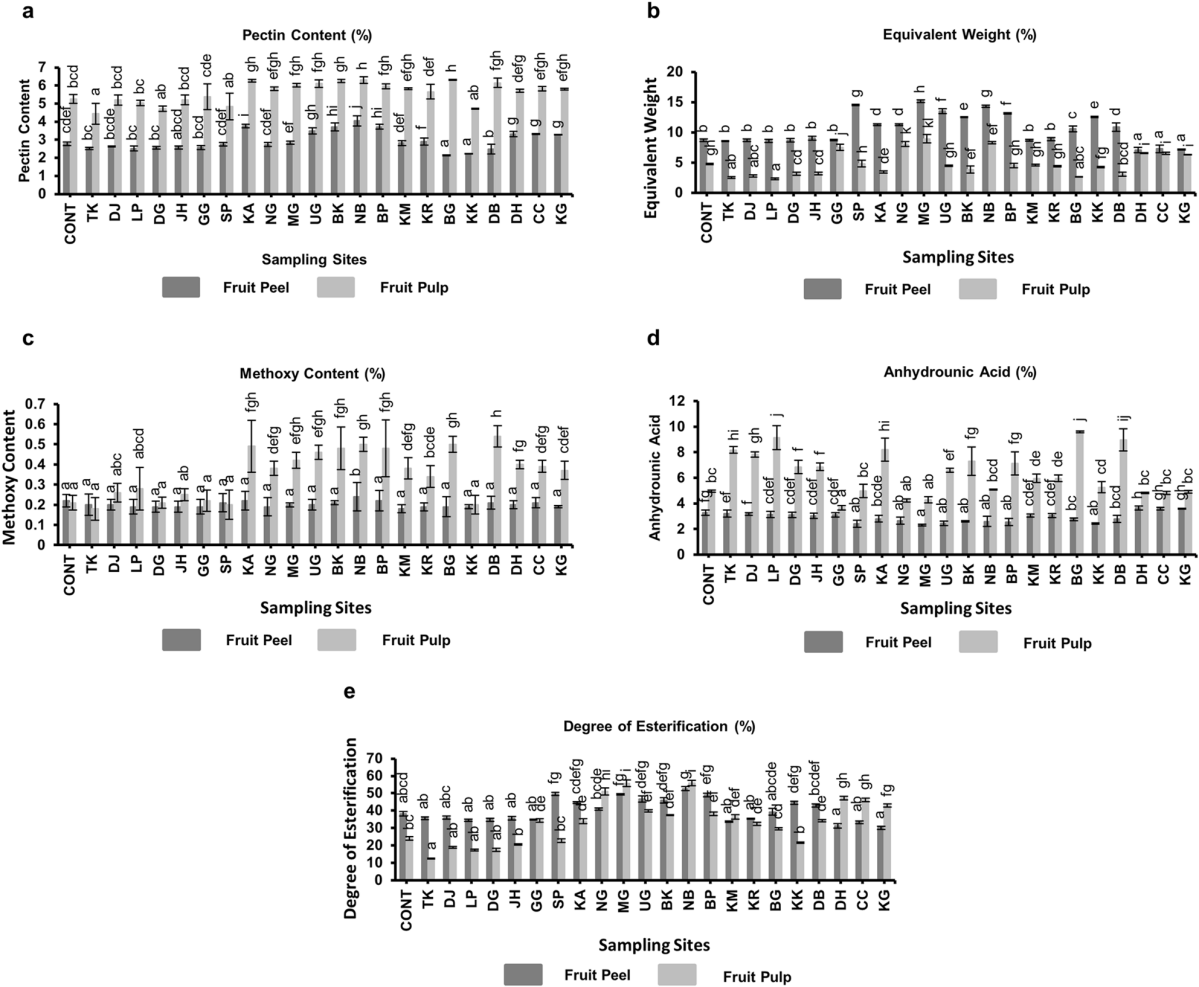
Chemical characterization of pectin obtained from Assam lemon fruit across different districts of Assam; (**a**)**:** Pectin Content, (**b**)**:** Equivalent Weight, (**c**)**:** Methoxy Content, (**d**)**:** Anhydrounic Acid, (**e**)**:** Degree of Esterification. Data represent mean values (column bars) with standard deviation of n = 3 biological replicates represented as vertical lines on the column bars. Different letters on top of the bars represent significant differences between samples at subset for alpha = 0.05, based on Duncan Multiple Range Test (DMRT) test.

The results of pectin estimation for the various districts in Assam along with the control population revealed that Nalbari district had the highest concentration of pectin content on the peel of Assam lemon fruit (4.05 ± 0.282%), while Bongaigaon had the lowest concentration (2.14 ± 0.02%). On the other hand, Bongaigaon had the highest concentration of pectin content in the pulp of Assam lemon fruit (6.32 ± 0.02%), while Udalguri had the lowest (4.44 ± 0.572%) (Fig. 15, Supplementary file 5).

#### Equivalent weight

The result of equivalent weight estimation for the various districts in Assam along with the control population showed that Sonitpur had the highest concentration of equivalent weight on the peel of Assam lemon fruit (15.17 ± 0.25%), while Dima Hasao had the lowest concentration (7.08 ± 0.447%). On the other hand, Morigaon had the highest concentration of equivalent weight in the pulp of Assam lemon fruit (8.96 ± 0.7%), while Lakhimpur had the lowest (2.31 ± 0.129%) (Fig. 15, Supplementary file 5).

#### Methoxy content (MeO)

The result of methoxy content estimation for the various districts in Assam along with the control population showed that Nalbari had the highest concentration of methoxy content on the peel of Assam lemon fruit (0.24 ± 0.027%), while Morigaon had the lowest concentration (0.18 ± 0.02%). On the other hand, Dhubri had the highest concentration of methoxy content in the pulp of Assam lemon fruit (0.54 ± 0.5%), while Tinsukia had the lowest (0.18 ± 0.06%) (Fig. 15, Supplementary file 5).

#### Anhydrounic acid (AUA)

The result of anhydrounic acid content estimation for the various districts in Assam along with the control population showed that Dima Hasao had the highest concentration of anhydrounic acid content on the peel of Assam lemon fruit (3.64 ± 0.15%), while Morigaon had the lowest concentration (2.3 ± 0.07%). On the other hand, Bongaigaon had the highest concentration of anhydrounic acid content in the pulp of Assam lemon fruit (9.59 ± 0.08%), while Tinsukia had the lowest (3.64 ± 0.17%) (Fig. 15, Supplementary file 5).

#### Degree of esterification (DE)

The results of degree of esterification for the 22 selected different districts of Assam along with control population revealed that only Nalbari (52.61 ± 1.172%) district exhibited a high degree of esterification (> 50%) in peel samples. In pulp samples, Nalbari, Morigaon, and Nagaon districts displayed the highest esterification levels at 55.95 ± 1.528%, 55.67 ± 1.924%, and 51 ± 1.978%, respectively. These findings emphasize the notable esterification concentrations in these districts’ peel and pulp samples compared to others, indicating potential variations in the composition of ester bonds in these regions (Fig. 15, Supplementary file 5).

### Analysis of variance (ANOVA)

During the investigation of variation of Assam lemon from different populations of Assam based on morphological and biochemical characters, we observed that there is a significant variation among the population of selected districts (*p* < 0.05), indicating a variation in the different morphological characters including seeding pattern and biochemical traits.

### Soil nutrient estimation

The soil nutrient assessment conducted for the current study revealed that the sampled populations exhibited a range from acidic to slightly alkaline. Most of the studied districts showed predominantly acidic soil, while populations from certain districts such as Lakhimpur, Golaghat, Sonitpur, Nagaon, Kamrup Metro, Kamrup Rural, Kokrajhar, and Dhubri displayed both acidic and alkaline pH levels. Interestingly, the control population and districts like Karbi Anglong, Dima Hasao, Cachar, and Karimganj showcased an alkaline soil nature.

The study also showed that the soil collected from all selected populations exhibited a significant presence of vital macro nutrients. The findings demonstrated that the soil in all populations of selected district contained a substantial amount (ranging from 0.505 to 1.5%) of Organic Carbon, except for the sites in Karbi Anglong, where the Organic Carbon concentration was comparatively lower (0.100–0.500%) compared to the populations of other districts. Moreover, the soil samples from the populations of Nagaon, and Dhubri along with the control population, displayed a mixed concentration of Organic Carbon, ranging from 0.100% to 1.500%. Furthermore, current study also revealed that the soil samples from all populations in the selected districts exhibit a notable concentration of phosphate, ranging from 22 to < 73 kg/ha, except for populations in Karbi Anglong district, which had phosphate levels > 22 kg/ha. In addition, the study findings revealed a significant presence of Potassium in the soil across most of the populations of selected districts, with concentrations ranging from 112 to 392 kg/ha, except for the populations in Tinsukia, Dibrugarh, Dhemaji, Jorhat, Golaghat, Sonitpur, Morigaon, Kamrup Metro, and Kokrajhar. In these districts, the Potassium concentration (> 112 kg/ha) was relatively lower. On the other hand, the soil samples from Lakhimpur, Nagaon, Kamrup Rural, Nalbari, Bongaigaon, Dima Hasao, and Cachar displayed varying concentrations from low to high level of Potassium in the soil, ranging from > 112 to 392 kg/ha. Additionally, the results show that, all the populations in the selected districts, including the control population, displayed lower concentrations of Ammoniacal Nitrogen (15 kg/ha) and Nitrate Nitrogen (4–10 kg/ha) in the soil (Supplementary file 6).

The study also revealed that in addition to macronutrients, the soil in different regions in the Assam is also abundant in various micronutrients (Copper, Zinc, Boron, Manganese, Iron, and Molybdenum) which plays a crucial role in the healthy growth and development of plants, even though they are required in smaller quantities compared to macronutrients. The findings demonstrated that the soil from all the selected districts contains a significant amount of Copper (ranging from 0.5 to > 2 ppm) except some populations of Cachar and Dhubri districts along with all the populations of Karimganj district, where Copper content in the soil is relatively lower (ranging from 0 to 0.5 ppm). It has also been noticed that the levels of Zinc in the soil samples are generally low, ranging from 0 to 0.5 ppm, except for specific areas in Tinsukia, Lakhimpur, and Cachar where the concentration of zinc was higher, ranging from 0.5 to 2 ppm. Again, Boron concentration in the soil samples exhibited significant variations across the sampled districts. Notably, Sonitpur, Nagaon, and Karbi Anglong districts displayed a significant presence (> 2 ppm) of Boron, whereas soil samples from the Cachar district and the control population showed considerably lower Boron levels (ranging from 1 to 2 ppm). In contrast, the soil samples of the remaining districts exhibited a diverse range of Boron concentrations, varying from 0.1 to > 2 ppm. A notable variation in Manganese concentration was observed among all the populations of selected districts, demonstrating high Manganese levels (> 4 ppm) in the accessions of Tinsukia, Dhemaji, Lakhimpur, Udalguri, Baksha, Nalbari, and Barpeta, while Sonitpur, Nagaon, Kokrajhar, and Karbi Anglong exhibited comparatively lower concentrations (ranging from 2 to 4 ppm) in contrast to the aforementioned districts in Assam. The concentration of Manganese were however found to be significantly low (0–2 ppm) in the populations of Dibrugarh, Jorhat, Golaghat, Morigaon, Kamrup Metro, Kamrup Rural, Bongaigaon, and the control population. Further, the soil samples from the populations of Dima Hasao, Cachar, Karimganj, and Dhubri displayed a diverse range of Manganese concentration, ranging from 0.2 to > 4 ppm. Additionally, the soil samples from Dhemaji, Lakhimpur, Dibrugarh, Jorhat, Golaghat, Sonitpur, Udalguri, Baksha, Nalbari, Barpeta, Karbi Anglong, Nagaon, Morigaon, Kamrup Metro, Kamrup Rural, and Karimganj along with the control population displayed a significant concentration (ranging from 3 to > 6 ppm) of Iron in the soil, whereas samples from all the populations in Dhubri were found to be significantly low in Iron concentration (ranging from 1 to 3 ppm). Further, the soil samples collected from Tinsukia, Dima Hasao, Cachar, and Kokrajhar exhibited a wide range of Iron concentrations, ranging from 1 to > 6 ppm. The concentration of Molybdenum however remained consistently low (ranging from 0 to 1 ppm) across all districts, including in the control population (Supplementary file 7).

## Discussion

Morphological characters analysis plays a crucial role in the investigation of plants, enabling researchers to explore the vast array of variations in a plant species^37^. By closely examining the morphological characteristics of plants, valuable insights can be gained not only into the diverse traits and variations within the different plant species, cultivars, and varieties but also within the same cultivar and variety across different regions^38–40^. The morphological variation observed among Assam lemon populations from different districts of Assam provides valuable insights into the morphological variation within the cultivar. During the current study, we observed that there is a significant variation in several morphological characters including fruit weight, fruit length, fruit diameter, pulp to peel ratio and others among the 132 populations from the 22 districts along with the control, thus indicating variation in important fruit characters of Assam lemon fruits. The ANOVA analysis revealed a statistically significant difference (*p* < 0.05) among the populations, indicating a notable variation in morphological characters^41^. This finding highlights the presence of distinct morphological variations within the Assam lemon cultivar across different populations. Similar investigation was also observed on *C. nobilis* Lour^42^, *Cynodon dactylon* (L.) Pers.^43^, *C. reticulate* Blanco^44^, which highlighted the differences in morphological traits across different populations. Interestingly, in the Upper Assam districts, excluding Golaghat, Assam lemon trees exhibit a flowering pattern wherein the prevalence of bisexual flowers surpasses that of unisexual flowers. Nevertheless, the proportion of unisexual flowers showed an increasing trend from Golaghat district towards Central, North, and Lower Assam, as well as Barak Valley, eventually reaching an almost equal concentration compared to bisexual flowers. This variation could be attributed to various factors, including environmental conditions, soil composition, and farmer practices specific to each region^7^.

The PCoA analysis of the population utilizing morphological characters provided compelling evidence of high morphological divergence among the Assam lemon populations. The results indicate presence of two distinct and well-defined groups that vary from each other in terms of their morphological traits. This emphasizes the significance of considering the morphological diversity within the Assam lemon cultivar, as it holds implications for genetic differentiation, adaptation, and potential breeding strategies^45^. These findings also open up avenues for further investigation into the underlying factors (such as environmental influences, genetic factors, or geographical factors) contributing to the observed morphological variations, thus can enhance our understanding of the complex dynamics within the Assam lemon populations^46^. Similar observations were also documented for UPGMA clustering analysis, where a total of 132 populations formed two distinct clusters. Notably, the second cluster displayed further subdivision into two sub-clusters. The UPGMA clustering analysis shows that the populations from Dhemaji and Tinsukia districts exhibit more morphological similarity with the control population than other districts. Interestingly, in our previous reported work (Ahmed et al. in 2023), we observed similar outcomes while investigating the genetic diversity of Assam lemon using ISSR marker system where populations from Dhemaji and Tinsukia districts showed close resemblance with the control population^4^. This observation could be attributed to relatively fewer mutations occurring over time in these populations compared to other populations studied. In contrast, populations from Kokrajhar, Bongaigaon, Dhubri, Cachar, and Karimganj exhibited significant morphological differentiation from the control population. Similar observations has been made in few other species such as *Cyclamen sp*.^47^, *Cichorium sp*.^48^, *C. Maxima* (Burm.) Merr.^49^. This implies that genetic variations or mutations could be one of the reasons for the observed variation in different characters in different regions of Assam^50^.

During the investigation of seed count among different populations in selected districts, we observed significant variation in the number of seeds. Specifically, the populations of Dhemaji, Tinsukia, Dibrugarh, Lakhimpur, and Jorhat, along with the control population, exhibited a seedless nature in their fruits which is its characteristic nature for which this cultivar was introduced and attained popularity^10^. However, the populations starting from Golaghat districts towards Central Assam, North Assam, Lower Assam and Barak Valley districts showed a mixed character, with some accessions having both forms and others were seedless fruits. Interestingly, the results of the seeding pattern resemble the variation in flowering nature. Assam lemon could be a self-incompatible cultivar having tendency towards natural hybridization^11–13^.

Additionally, the number of seeds found in Assam lemon fruits across different accessions was studied. The study observed variations in seed counts among different accessions, with specific patterns emerging in different districts with variations ranging from no seeds to more than 10 seeds. The variations in seed counts among different accessions of Assam lemon fruits, coupled with specific patterns observed in different districts, highlights the complexity of factors influencing seed pattern. It was previously reported that the environmental factors and genetic mutations act as a potential influencer on the seeding pattern of fruits^51^. Also, the variation in the soil nutrients in different districts of Assam may be a reason, which contributes to the differences in seeding patterns among the accessions in the current study^50^. Furthermore, the inherent propensity of *Citrus* species, including Assam lemon, for natural hybridization could potentially account for variations in seed formation and seed counts among different populations over time^52^. Given the contemporary emphasis on seedless fruit in response to consumer preferences, modern farmers and breeders are actively engaged in the development of diverse seedless fruit varieties^15^. The investigation into the variations in the seeding pattern of Assam lemon holds the potential to significantly impact cultivation techniques, aligning with consumer desires for enhanced fruit quality with seedlessness being a desired trait^53,54^.

Apart from the above-mentioned observations, variation in different biochemical parameters was found in the fruits of Assam lemon across the selected districts. A significant variation in pH concentration was observed in the samples from Karbi Anglong, Sonitpur, Baksha, Udalguri, Morigaon, Dhubri, and the Barak Valley districts (Dima Hasao, Cachar and Karimganj) where the pH was shown to be higher (> 2.5) as compared to other districts and the control population. The change in pH may be due to the variation in agro-environmental factors, such as soil nutrient composition, rainfall, and temperature, which can influence pH levels in fruits^55^. The current investigation also revealed that the samples from Nagaon, Kamrup Metro, and Kamrup Rural districts exhibited higher concentrations of TSS (≥ 8) compared to other districts and the control population, which is an important biochemical parameter used in various processing applications, such as juicing, canning, and making jams, jellies, and others^56^. The high TSS concentrations indicate an increased content of dissolved sugars, organic acids, and other soluble compounds in Assam lemon samples of Nagaon, Kamrup Metro, and Kamrup Rural districts^57^. Environmental conditions, including sunlight, rainfall, temperature, soil nutrients, and agricultural practices have been reported to affect TSS values in fruits^58^. These factors could be the reason for variation in TSS concentrations observed across different regions of Assam^58^.

Assam lemon is also rich in juice content as compared to other different lemon varieties due to its large size^5^. During the investigation we found that there is a significant variation in the % of juice content among different regions of Assam. All the districts along with the control population exhibited % juice content below 50%, except for Morigaon, Karbi Anglong, and the districts of the Barak Valley, which displayed a juice content exceeding 50%. Interestingly, the results also revealed that, although the populations from Dhemaji and Tinsukia districts exhibited resemblances with the control population in terms of morphological and other biochemical attributes, a notable difference emerged in terms of % juice content. Specifically, the population from the Dhemaji district displayed a lower % juice content (< 40%) at 32.13 ± 0.036% in comparison to both the control population and the Tinsukia district population (> 40%) at 41.24 ± 0.071% and 45.92 ± 0.92% respectively. The observed variations in juice content across different regions can be attributed to a range of regional factors, such as climate, soil composition, agricultural practices, and even genetic variations^59,60^. Further, during the current investigation, an interesting observation regarding the concentration of citric acid in Assam lemon across different districts was noted, it was observed that the districts of Dhemaji, Tinsukia, and Dibrugarh in Upper Assam and the control population had the lowest concentration of citric acid. However, as we moved towards Jorhat and Golaghat, the concentration of citric acid gradually increased. The districts in the Barak Valley exhibited the maximum concentration of citric acid. This variation in citric acid content suggests differences in the enzymatic activity responsible for citric acid synthesis during fruit development^61^. In addition to citric acid, we also examined the ascorbic acid content in the Assam lemon samples. Surprisingly, it was found that all the districts, along with the control population, had ascorbic acid content below 0.4 mg/ml, except for the samples from the Dhubri district, which displayed an ascorbic acid content of 0.607 mg/ml. The variation in acid content among the Assam lemon fruits could be attributed to environmental conditions, including temperature, sunlight exposure, rainfall, and other factors^62^. It has been reported that the ascorbic acid content of *Citrus* fruits is not stable and can vary due to enzymatic loss, where l-ascorbic acid is converted to 2–3-deoxy-l-gluconic acid^63^. Additionally, the utilization of ascorbic acid in metabolic processes might contribute to its decrease during the maturation process^64^. On the other hand, it was observed that the fruits obtained from Dhemaji, Tinsukia, Dibrugarh, Jorhat, and Golaghat, as well as Kamrup Metro and Kamrup Rural districts, exhibited high level of TSS/TA compared to samples from other districts. Fruits from these regions displayed higher levels of TSS and a significant decrease in acid accumulation, which could be the contributing factors to the decline in the TSS/TA ratio^61^. Further, during the current investigation of Assam lemon fruit juice, a noteworthy finding emerged regarding the relationship between sugar concentration and pH, we observed a negative correlation between these two variables across most of the samples analyzed. This implies that as the sugar concentration increased, there was a corresponding decrease in the pH of the juice^65^. This intriguing pattern might be due to the accumulation of organic acids within the juice^66^. These organic acids not only impact the sugar concentration but also play a crucial role in determining the overall pH of the fruit juice^67^. Hence, the observed variations in sugar concentration may be attributed to the influence of these organic acids, which subsequently affect both the sugar concentration and the pH levels of the juice^66,67^. Additionally, while studying the carotenoid and chlorophyll content in samples of selected districts along with the samples from control population, a negative correlation between the carotenoid and chlorophyll content was observed. Low chlorophyll levels and high carotenoid levels may be favored by specific environmental conditions that promote carotenoid synthesis and accumulation^68^. The environmental factors such as light intensity, temperature, and nutrient availability may be the causes which influenced the pigment composition of Assam lemon peel^69^.

Pectin, a crucial ingredient used in various food products, including jams, jellies, and low-calorie foods, was found to be abundant in both the peel and pulp of the selected samples from different districts, including the control population^70^. However, it was noted that the pectin concentration was consistently high in the pulp compared to the peel. Interestingly, when comparing the pectin concentration among districts, it was found that samples from Upper Assam and the control population exhibited lower pectin concentrations compared to samples from other districts. Environmental conditions and genetic mutations might be the attributing factors to these variations, which have also been previously reported to influence pectin production^71,72^. Pectin can be categorized as either high-methoxy pectin or low-methoxy pectin based on its esterification extent^33^. High-methoxy pectin is known for its sensitivity to acid conditions and typically requires a substantial amount of sugar to function as a thickening and gelling agent^73^. On the other hand, low-methoxy pectin has gained prominence in the food industry, particularly for jam production, as it can form a gel with less reliance on sugar^73^. To ensure the purity of the extracted pectin, we also evaluated the anhydrounic acid content in both the peel and pulp of Assam lemon samples^33^. Notably, the pulp displayed the highest anhydrounic acid content, followed by the peel. Findings from the current study indicated that samples from the control population, Upper Assam, Barak Valley, Bogaigaon, Kokrajhar, and Dhubri districts have highest pectin purity compared to samples from other districts. The present investigation also delved into the esterification level of pectin, specifically focusing on the concentration of methylesters, which plays a significant role in determining the applicability of pectin in various food industries^74^. Notably, the peel samples from Nalbari and the pulp samples from Morigaon and Nalbari exhibited high concentrations of methylesters, indicating the presence of high-methoxy pectin. In contrast, the samples from all other districts, including the control population, displayed low concentrations of methylesters, suggesting the prevalence of low-methoxy pectin. Factors such as climate, soil composition, and agricultural practices specific to each region may contribute to these variations^55^. These factors may collectively influence the physiological characteristics of Assam lemon trees, leading to variations in fruit development and juice production^45,75^.

The soil composition and nutrient availability play a pivotal role in shaping the morphological characters of plants, including various fruit traits such as length, diameter, peel thickness, seed number, and more^76^. During the current investigation, soil nutrient analysis from 132 populations revealed a significant variation not only in the nutrient content but also in the soil nature itself. This variation in soil characteristics is likely to be a crucial factor contributing to the observed variations in different morphological characters of Assam lemon within the selected populations^77^. It’s important to note that soil fertility and nutrient content can vary widely based on geographical and environmental factors. Therefore, regular soil testing and analysis are essential for farmers to make informed decisions regarding nutrient management and crop production.

By catering to demands for high yield, substantial juice content, and the absence of seeds, the outcomes of this study can play a pivotal role in guiding cultivation practices and meeting the evolving expectations of consumers^54^. Thus, the findings of this study have practical implications for growers and breeders as they can select suitable populations with desired morphological characters for commercial cultivation and breeding programs.

## Conclusion

The present investigation underscores distinct variations in morphological, seeding patterns, and biochemical traits among Assam lemon accessions, with a notable affinity of Upper Assam districts to the control. Remarkably, the Upper Assam population demonstrates a noteworthy resemblance to the control, particularly in terms of its flowering characteristics. This includes the presence of both bisexual and unisexual flowers, with less concentration of unisexual flowers. Moreover, the seed availability pattern in these populations is notable, featuring the development of seedless Assam lemon fruits. In contrast, populations from other selected districts exhibited both bisexual and unisexual flowers with nearly equal concentrations. Additionally, these populations harbour both seeded and seedless fruits. The identified variation in the seeding pattern of Assam lemon holds promising potential to significantly influence cultivation practices, meeting consumer preferences for seedless fruits and improved quality. Further, biochemical analyses unveiled significant variation across districts, although populations from Dhemaji, Tinsukia, Lakhimpur, Dibrugarh, and Jorhat demonstrated similarity with the control. These insights contribute to our understanding of the cultivar’s diversity and hold implications for future breeding programs. By catering to the growing demands for high yield, substantial juice content, nutritionally rich and the absence of seeds, the findings of this study carry substantial importance in shaping cultivation practices. Thus, the findings of this study have practical implications for growers and breeders as they can select suitable populations with desired morphological characters for commercial cultivation and breeding programs.

### Supplementary Information

Supplementary Legends.

Supplementary Table S1.

Supplementary Table S2.

Supplementary Table S3.

Supplementary Table S4.

Supplementary Table S5.

Supplementary Table S6.

Supplementary Table S7.

## Data Availability

Data is contained within the article and the supplementary files.
