# Supplementary material for: Evaluation of morphological traits, biochemical parameters and seeding availability pattern among Citrus limon ‘Assam lemon’ accessions across Assam
Source: Sci Rep. 2024 Feb 16;14:3886. doi: 10.1038/s41598-024-54392-3 (PMC10873318; doi:10.1038/s41598-024-54392-3)
Supplement: Supplementary file 1 — Supplementary Legends. [file 41598_2024_54392_MOESM1_ESM.docx]

**Supplementary File Legends:**

Table SF **1:** Details of Assam lemon accessions collected from different districts of Assam

Table SF **2:** Variation in morphological characters of Assam lemon accessions across different districts of Assam

Table SF 3: Variation in flowering characters of Assam lemon accessions across different districts of Assam. Numerical data represent mean values (column bars) with standard deviation of n = 3 biological replicates represented as vertical lines on the column bars. Different letters on top of the bars represent significant differences between samples at subset for alpha = 0.05, based on Duncan Multiple Range Test (DMRT) test

Table SF **4:** Variation in fruit characters and seed characters of Assam lemon accessions across different districts of Assam. Numerical data represent mean values (column bars) with standard deviation of n = 3 biological replicates represented as vertical lines on the column bars. Different letters on top of the bars represent significant differences between samples at subset for alpha = 0.05, based on Duncan Multiple Range Test (DMRT) test

Table SF **5:** Variation in biochemical traits of Assam Lemon across different districts of Assam. Numerical data represent mean values (column bars) with standard deviation of n = 3 biological replicates represented as vertical lines on the column bars. Different letters on top of the bars represent significant differences between samples at subset for alpha = 0.05, based on Duncan Multiple Range Test (DMRT) test

Table SF **6:** Estimation of micronutrients from the Assam lemon population grown soil across different districts of Assam

Table SF **7:** Estimation of macronutrient from the Assam lemon population grown soil across different districts of Assam
